# Near Room-Temperature Intrinsic Exchange Bias in an Fe Intercalated ZrSe_2_ Spin Glass

**DOI:** 10.1021/jacs.3c06967

**Published:** 2023-08-30

**Authors:** Zhizhi Kong, Corey J. Kaminsky, Catherine K. Groschner, Ryan A. Murphy, Yun Yu, Samra Husremović, Lilia S. Xie, Matthew P. Erodici, R. Soyoung Kim, Junko Yano, D. Kwabena Bediako

**Affiliations:** † Department of Chemistry, University of California, Berkeley, California 94720, United States; ‡ Molecular Biophysics and Integrated Bioimaging Division, Lawrence Berkeley National Laboratory, Berkeley, California 94720, United States; § Chemical Sciences Division, Lawrence Berkeley National Laboratory, Berkeley, California 94720, United States

## Abstract

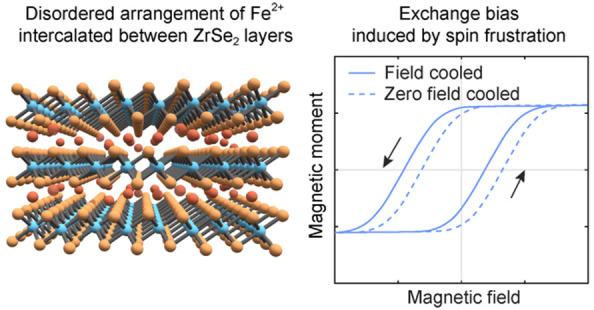

Some magnetic systems display a shift in the center of their magnetic hysteresis loop away from zero field, a phenomenon termed exchange bias. Despite the extensive use of the exchange bias effect, particularly in magnetic multilayers, for the design of spin-based memory/electronics devices, a comprehensive mechanistic understanding of this effect remains a longstanding problem. Recent work has shown that disorder-induced spin frustration might play a key role in exchange bias, suggesting new materials design approaches for spin-based electronic devices that harness this effect. Here, we design a spin glass with strong spin frustration induced by magnetic disorder by exploiting the distinctive structure of Fe intercalated ZrSe_2_, where Fe(II) centers are shown to occupy both octahedral and tetrahedral interstitial sites and to distribute between ZrSe_2_ layers without long-range structural order. Notably, we observe behavior consistent with a magnetically frustrated and multidegenerate ground state in these Fe_0.17_ZrSe_2_ single crystals, which persists above room temperature. Moreover, this magnetic frustration leads to a robust and tunable exchange bias up to 250 K. These results not only offer important insights into the effects of magnetic disorder and frustration in magnetic materials generally, but also highlight as design strategy the idea that a large exchange bias can arise from an inhomogeneous microscopic environment without discernible long-range magnetic order. In addition, these results show that intercalated TMDs like Fe_0.17_ZrSe_2_ hold potential for spintronic technologies that can achieve room temperature applications.

## Introduction

In a system that displays magnetic hysteresis, the magnetization depends on the history (sweep direction) of the external magnetic field, produces a remanent magnetization at zero-field, and a nonzero coercive field is needed to reestablish a zero-magnetization state following polarization.^1^ Magnetic hysteresis loops are usually centered on zero-field. However, some systems display a shift in the center of the hysteresis loop away from zero field—usually when cooled under an external magnetic field—an effect that is termed “exchange bias”.^2,3^ Magnetic materials that exhibit this exchange bias effect play a crucial role in spin-based memory and electronic devices (including spin valves,^4,5^ magnetic read heads,^6^ and magnetic random access memory^7^), since the shifted hysteresis loop prevents random noise from inadvertently reversing the magnetization of a component. Traditionally, magnetic heterostructures and thin films have been the primary platforms for engineering and studying exchange bias—including ferromagnetic (FM)/antiferromagnetic (AFM) bilayers^8−12^ (e.g., NiFe/FeMn), FM/ferrimagnetic (FiM) architectures^13,14^ (e.g., NiFe/TbCo), and FiM/AFM heterostructures^15^ (e.g., Fe_3_O_4_/CoO). Yet, despite the extensive study of exchange bias within a variety of such systems over seven decades, a comprehensive mechanism behind exchange bias remains lacking. One key unresolved issue is an understanding of the critical role of disorder and spin frustration at the interfaces of the constituent layers.

In canonical exchange bias constructs comprising layers of a FM on an AFM, the traditional explanation of exchange bias involves interfacial pinning of the FM layer to the adjacent AFM layer, resulting in a hysteresis loop that is polarized with the orientation of the AFM moment at the interface.^16^ This simplistic picture has since been replaced by a model in which exchange bias is driven by a disordered magnetic state—a spin glass (SG)—that is incidentally formed at the interface of the FM and AFM layers. This SG, which is characterized by random and frustrated exchange interactions, is believed to stem from the interplay of structural disorder,^12,17,18^ interface roughness,^9,19,20^ chemical intermixing^21^ and/or opposing, yet energetically similar, exchange interactions.^22^ Several studies have suggested that the spin glass itself is indeed closely tied to the exchange bias phenomenon, since this effect has been observed not only in prototypical magnetic heterostructures including FM/SG bilayers^18^ but also in apparently intrinsic single phase spin glass materials, including magnetically glassy dilute metal alloys,^23^ single crystals of compounds with inherently coexisting magnetic phases of AFM and SG,^24^ and geometrically frustrated lattices.^25^ Still, the role of magnetic disorder and frustration on the emergence of exchange bias remains unclear, though evidently critical for optimizing material and device performance.^26^

Layered transition metal dichalcogenides (TMDs) are a class of materials in which two-dimensional layers of *MCh*_2_ (*M* = transition metal; *Ch* = S, Se, Te) are stacked via van der Waals (vdW) interactions along the crystallographic *c*-axis. The vdW interfaces allow for the intercalation of a range of chemical species, such as atoms,^27^ molecules,^28^ and ions.^29−31^ TMDs intercalated with open-shell first-row transition metals, *T*, are versatile platforms for designing magnetic materials, where the spin density and magnetic ordering can be precisely controlled through the choice of the host lattice, intercalant, and stoichiometry of the resulting *T*_*x*_*MCh*_2_ compound. When *x* = 1/4 or *x* = 1/3, it is possible for intercalants to fully order with commensurate superlattices (of size 2*a* × 2*a* or √3*a* × √3*a*, respectively, where *a* is the lattice constant of the primitive *MCh*_2_ lattice) and to exhibit long-range magnetic order.^32−34^ However, when the extent of intercalation deviates from these stoichiometric compositions, some *T*_*x*_*MCh*_2_ materials have been found to exhibit spin-glass phases.^24,35,36^ Analytis and colleagues recently synthesized slightly off-stoichiometric Fe_0.33±δ_NbS_2_ (δ ≤ 0.03), which displayed a predominant AFM order coexisting with a minor spin-glass phase, exhibiting a large exchange bias below 40 K.^24^ Interestingly, the spin glass in this material, though a very minor component, may also enable ultralow current-induced switching of the AFM order, a technology that holds promise for low power spintronics.^31,32^ Intercalant site disorder coupled with the oscillatory nature of the purported Ruderman–Kittel–Kasuya–Yosida (RKKY) exchange interaction (i.e., exchange mediated by conduction electrons) was proposed as the origin of the crucial spin glass phase. In turn, the coupling between an uncompensated spin glass and a highly anisotropic AFM within a single crystal was suggested as the source of the highly enhanced exchange bias effect.

**Figure 1:**
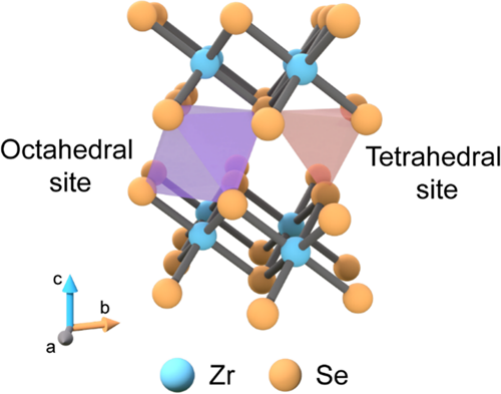
Intercalants can occupy octahedral and tetrahedral interstitial sites in 1*T*-ZrSe_2_.

To directly interrogate the role of intercalant disorder on spin-glass behavior in intercalated TMDs, we sought to explore a system possessing the spin glass itself as the predominant magnetic component. To accomplish this, we chose Fe_*x*_ZrSe_2_ for three reasons. First, structurally, ZrSe_2_ is a promising intercalation host lattice for constructing a predominant spin glass phase that may enable the study of an intrinsic spin glass-derived exchange bias effect. While the 2*H* and 1*T* polytypes of TMDs contain both octahedral and tetrahedral vacancy sites in the interlayer vdW sites, the experimentally determined crystal structures of most Fe-intercalated TMDs compounds show that intercalants occupy the octahedral sites exclusively.^37^ However, Fe-intercalated Zr-based TMDs (Fe_*x*_Zr*Ch*_2_; *Ch* = S, Se) are unique in that intercalants can occupy both tetrahedral and octahedral sites (Figure 1), with Fe atoms distributed between both sites.^38,39^ The difference in coordination environment of intercalated Fe at different sites, combined with the variation in the distance between magnetic sites and the oscillatory nature of potential exchange interactions (including p–d hybridization for semiconductors,^40,41^ RKKY for metals^42−44^), should lead to variations in sign and magnitude of the coupling between adjacent magnetic centers, potentially resulting in spin frustration that might be expected to manifest as a spin-glass phase. Second, the development of systems with high transition temperatures closer to room temperature is crucial for any potential technological application. In this regard, selenide analogues of intercalated TMDs are promising, as these compounds generally exhibit higher magnetic ordering temperatures compared to their related sulfides (likely due to the stronger spin–orbit coupling in heavier selenide compounds and/or greater orbital overlap).^38,39,45−47^ Finally, Fe_*x*_ZrSe_2_ has been reported to be semiconducting.^39^ The interaction of any localized moments with conduction band electrons has a profound effect on exchange interactions. To a greater extent than metals, semiconducting materials permit the strong modulation of their charge carrier densities electrostatically^48−50^ or electrochemically,^51^ offering the possibility of achieving electrically controlled magnetism.

As a consequence of the inherent intercalant disorder, we expect Fe_*x*_ZrSe_2_ to exhibit spin glass properties. While there have been suggestions that the material displays glassy magnetic behavior,^39^ the unambiguous experimental verification of this spin-glass state has not yet been reported, and the resultant manifestation of exchange bias has not been demonstrated. Here, we show that Fe_*x*_ZrSe_2_ (*x* ∼ 0.17) consists of high-spin Fe^2+^ ions located without long-range order in both octahedral and tetrahedral sites between the layers of 1*T*-ZrSe_2_. This material displays semiconducting behavior with a bandgap of about 0.4 eV, and magnetic characterization reveals a high degree of spin frustration above room temperature, much higher than the glassy transition temperatures (<40 K) reported for other Fe_*x*_*MCh*_2_ materials. Consequently, upon magnetic field cooling, Fe_*x*_ZrSe_2_ displays a measurable, apparently intrinsic, exchange bias for temperatures ≤250 K. These results indicate that the spin-glass phase is closely linked to the exchange bias phenomenon and suggest a powerful strategy for designing novel exchange bias systems with near-room temperature transition temperatures, which could have potential applications in future spintronics devices.

## Results

### Synthesis, Composition, and Structure of Fe-Intercalated ZrSe_2_

Single crystals of Fe_*x*_ZrSe_2_ were synthesized by using chemical vapor transport (CVT). A mixture of source materials Fe, Zr, and Se in a ratio of 0.5:1:2 with transport agent I_2_ (1 mg/cm^3^) was loaded into an evacuated quartz ampoule and placed in a two-zone furnace with temperature set points of 900 and 1050 °C (see the Supporting Information for additional experimental details and Figure S1 for setup). After 15 days, hexagonal plate-shaped silver-black crystals with lateral dimensions of several millimeters were obtained (Figure S1c). The crystals were thoroughly rinsed with toluene to remove the excess I_2_ and stored in an argon-filled glovebox.

**Figure 2:**
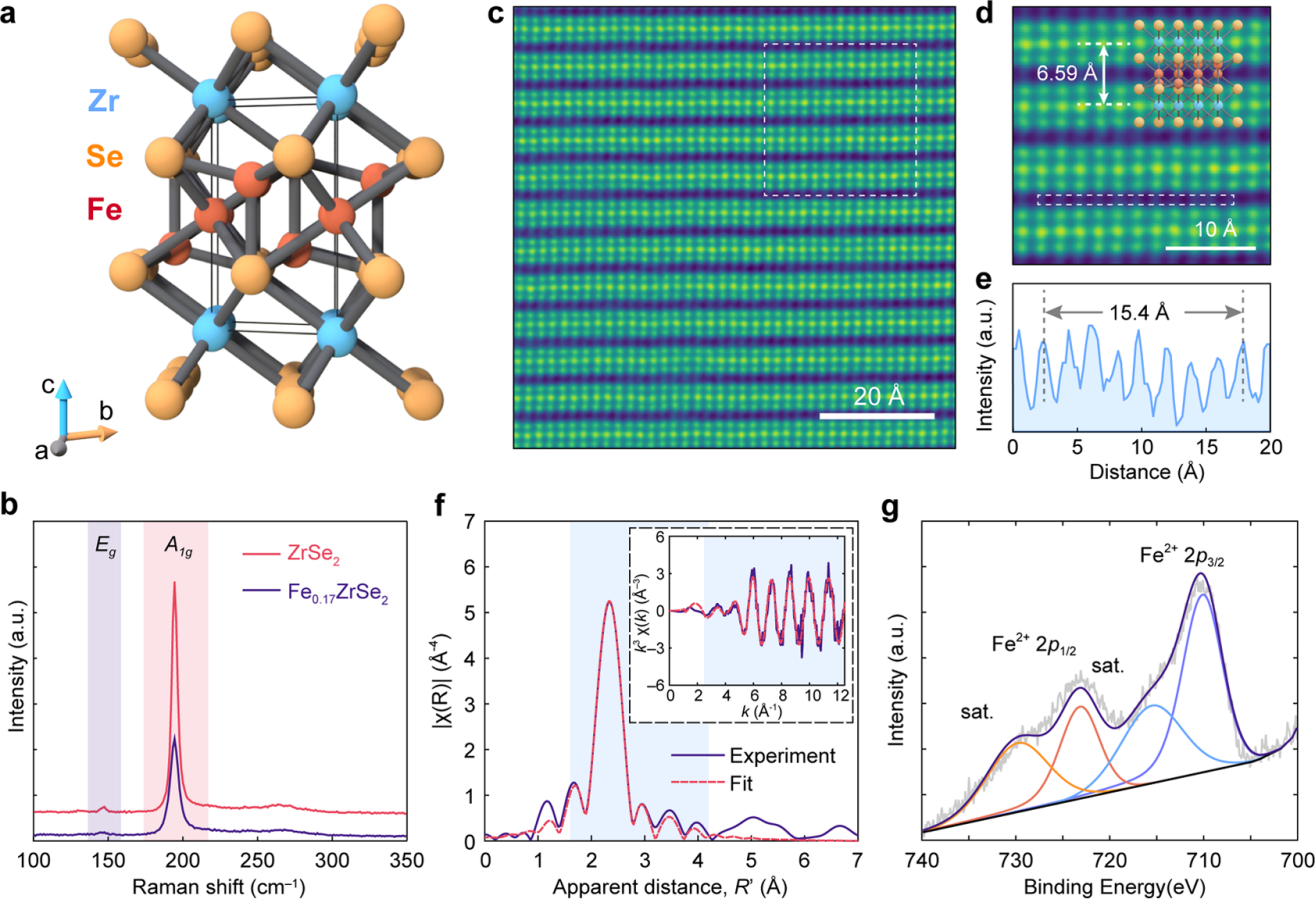
Compositional and structural characterization of Fe_0.17_ZrSe_2_. (a) Crystal structure of Fe_0.17_ZrSe_2_ is that of 1*T*-ZrSe_2_ with iron atoms intercalated in both octahedral and tetrahedral sites in the vdW interface. The occupancies of octahedral and tetrahedral sites are 0.1 and 0.07, respectively. The unit cell is framed in solid lines. (b) Raman spectra of 1*T*-ZrSe_2_ (red line) and Fe_0.17_ZrSe_2_ (purple line) flakes with 532 nm excitation. (c) Cross-sectional HAADF-STEM image of an Fe_0.17_ZrSe_2_ sample along the [101̅0] zone axis. (d) Zoomed-in image of white dashed line area in c overlaid with the Fe_0.17_ZrSe_2_ structure. (e) Line profile integrating intensity along the white dashed box in d revealing the presence of intercalant ions between the layers of 1*T*-ZrSe_2_. (f) Fe *K*-edge EXAFS spectra obtained at room temperature in a He atmosphere. Experiments are fitted by means of the ARTEMIS program. The figures show Fourier transforms |χ(*R*)| of the *k*^3^-weighted EXAFS spectrum (not corrected for phase shift). The inset shows the *k*^3^-weighted background-subtracted EXAFS spectrum. Experimental data is plotted as a solid purple trace and fits are shown as dashed red lines. The regions highlighted in blue are the fitting regions. (g) Fe 2p XPS spectrum in Fe_0.17_ZrSe_2_. The Fe 2p_3/2_ and Fe 2p_1/2_ features located at 710.09 eV (purple) and 723.15 eV (red), respectively, are attributed to Fe^2+^ centers. The peaks located at 715.5 eV (blue) and 729.8 eV (orange) are attributed to the Fe satellite peaks.

To determine the composition of Fe_*x*_ZrSe_2_ crystals, scanning electron microscopy (SEM) combined with energy-dispersive X-ray spectroscopy (EDS) was performed (see Supporting Information for experimental details). Prior to SEM-EDS measurements, the as-grown crystals were cleaved with adhesive tape to expose a fresh surface. The SEM image of the as-grown crystal and the corresponding EDS spectrum and elemental mapping are shown in Figure S2. The elemental maps are consistent with a uniform spatial distribution of Fe, Zr, and Se over a large area of approximately 90 000 μm^2^ with the Fe, Zr, and Se in the ratio 0.17:1:2.02. This composition is consistent with the empirical formula Fe_0.17_ZrSe_2_ determined through single-crystal X-ray diffraction (SCXRD) refinement (Table S1). The main X-ray diffraction peaks can be well integrated by using the trigonal space group (*P*3̅*m*1). The lattice parameters (*a* = *b* = 3.7662(2) Å, *c* = 6.1236(4) Å) at room temperature were found to be close to those of reported structures for ZrSe_2_,^52−55^ indicating that the intercalated Fe atoms did not significantly affect the interlayer distance. Figure 2a depicts the unit cell of the single-crystal structure solution, where the ZrSe_2_ host lattice is preserved in the CdI_2_ (1*T*) structure with Fe atoms partially occupying both tetrahedral and octahedral sites coordinated by Se atoms within the interlayer region.

Figure 2b presents the Raman spectra of ZrSe_2_ and Fe_0.17_ZrSe_2_ crystals obtained using 532 nm excitation. ZrSe_2_ exhibits the characteristic phonon modes, out-of-plane *A*_1g_ (194 cm^–1^) and in-plane *E*_g_ (147 cm^–1^) vibrations, consistent with previously reported values.^56^ The spectral features observed in Fe_0.17_ZrSe_2_ were found at the same Raman shifts as those of ZrSe_2_, providing additional evidence that the intercalation of Fe atoms did not break the crystal symmetry of host lattice 1*T*-ZrSe_2_.^57^ No Raman peaks corresponding to a superlattice were detected in Fe_0.17_ZrSe_2_, suggesting that Fe centers are disordered between the layers and no long-range superstructure is formed.^57^ The absence of superlattice formation is also supported by selected area electron diffraction (SAED), as only diffraction spots corresponding to the host lattice were observed (Figure S3). Furthermore, the decrease in peak intensities and the broadening of peak widths observed in the Raman spectrum of Fe_0.17_ZrSe_2_ are attributed to the site disorder induced by the disordered distribution of Fe centers in the lattice.^57,58^

To more directly establish the presence of Fe between ZrSe_2_ layers, we performed high angle annular dark-field scanning transmission electron microscopy (HAADF-STEM) of cross-sectional samples. Figures 2c, d shows HAADF-STEM cross-sectional images taken along the [101̅0] direction, revealing high crystallinity of the ZrSe_2_ lattice. Inspection of these HAADF-STEM images shows significant intensity between ZrSe_2_ layers, and Figure 2e displays a line intensity profile along the dashed rectangle in Figure 2d, confirming that substantial atomic contrast is present between the layers of ZrSe_2_, consistent with intercalated Fe centers.

The local electronic and geometric features of Fe centers in Fe_0.17_ZrSe_2_, were revealed with X-ray absorption spectroscopy, XAS (including X-ray absorption near-edge structure, XANES, and extended X-ray absorption fine structure, EXAFS) and X-ray photoelectron spectroscopy (XPS) on Fe_0.17_ZrSe_2_ crystals. Fitting to the EXAFS of the Fe_0.17_ZrSe_2_ crystals (Figure 2f) revealed that a majority of Fe atoms occupy octahedral positions, with a minority occupying tetrahedral sites. Paths were generated from the crystal structure of Fe_*x*_ZrSe_2_ (Figure 2a) where Fe atoms were placed in either the tetrahedral or octahedral positions. For each fit, paths corresponding to Fe–Se and Fe–Fe single-scattering for both tetrahedral and octahedral sites were included. There is minimal signal beyond *R* = 2.8 Å, shorter than all Fe–Fe and Fe–Zr paths, suggesting no clustering of Fe atoms near each other. No viable fits were obtained when any Fe–Zr scattering was included. To evaluate the ratio of octahedral to tetrahedral sites, the amplitude factor in the fits for the octahedral and tetrahedral paths was weighted by a factor *x* or 1–*x*, respectively, where *x* = 1 corresponds to 100% of Fe atoms in octahedral sites and *x* = 0 corresponds to 100% in tetrahedral sites. In a given fit, *x* was constant, and a series of fits with the same parameters were performed where *x* was varied in steps of 0.05 or 0.1 from 0 to 1. The best fit corresponds to 90% of the Fe atoms modeled in octahedral sites (see the Supporting Information, Section S7, Figure S5 for details).

XPS spectra of Fe_0.17_ZrSe_2_ single crystals (Figure 2g) are also consistent with Fe in the +2 oxidation state, displaying peaks at 710.09 eV (2p_3/2_) and 723.15 eV (2p_1/2_), which are consistent with what have been reported for Fe^2+^ in Se-based coordination environments.^59,60^ The comparison of Fe *K*-edge XANES spectra for Fe_0.17_ZrSe_2_ and the standard Fe-based compounds in Figure S4b also confirms the oxidation state of intercalated Fe atoms to be +2.

### Bandgap and Electronic Properties

**Figure 3:**
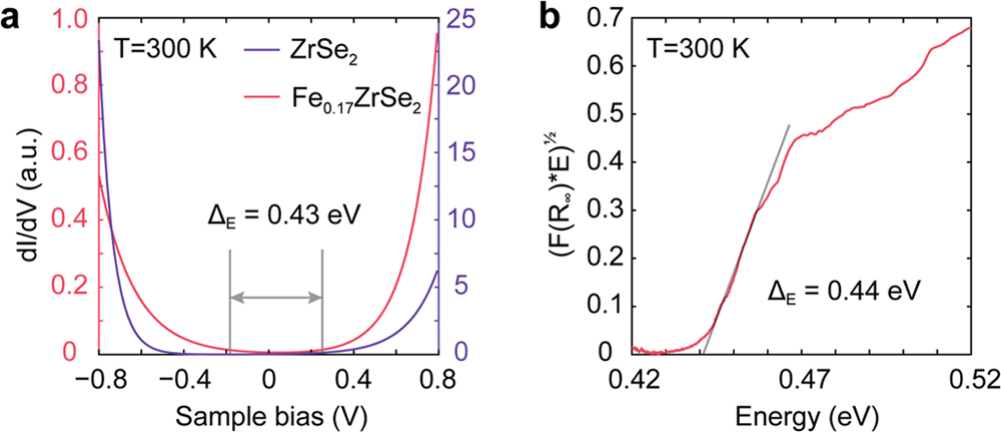
Bandgap characterization of Fe_0.17_ZrSe_2_. (a) Scanning tunneling spectra of the ZrSe_2_ crystal (purple line) and the Fe_0.17_ZrSe_2_ crystal (red line) at room temperature. (b) Tauc plot of the Fe_0.17_ZrSe_2_ crystal. The linear part of the plot is extrapolated to the *x*-axis. The value of the absorption spectrum *F*(*R*_∞_) was transformed from the raw diffuse reflectance spectrum by applying the Kubelka–Munk function.

The electronic properties of the bulk Fe_0.17_ZrSe_2_ crystal were characterized using scanning tunneling spectroscopy (STS) and diffuse reflectance spectroscopy (DFS) at room temperature (Figure 3). STS measurements were performed on a freshly cleaved bulk sample in ambient conditions. The sample was mounted into the STS sample holder using carbon tape to attach the top of the sample to an STS probe (see the Supporting Information, Section S9, Figure S6a for details). At a fixed tip–sample separation, the tunneling current between the tip and the Fe_0.17_ZrSe_2_ crystal was monitored, while the bias voltage (V) was swept over a given range. In this measurement, *V* modulates the position of the Fermi level within the electronic bands, which is manifest as a variation in the tunneling current, *I*, and therefore also a modulation in differential conductance, d*I*/d*V* (the instantaneous slope of the *I–V* trace). When the Fermi level lies within the bandgap, *I* and d*I*/d*V* approach zero, and nonzero *I* and d*I*/d*V* values are expected when the Fermi level lies within the valence or conduction band. Figure 3a shows d*I*/d*V* tunneling spectra of ZrSe_2_ and Fe_0.17_ZrSe_2_ determined from this STS measurement (Figure S6b-e). The flat region of d*I*/d*V* ≅ 0 near the origin corresponds to the band gap,^61^ revealing a band gap of approximately 0.43 eV. The comparison between STS for ZrSe_2_ and Fe_0.17_ZrSe_2_ shows that the intercalation of Fe into ZrSe_2_ substantially decreases the size of the band gap. Likewise, analysis of the DFS Tauc plot of Fe_0.17_ZrSe_2_ yields a band gap of ∼0.44 eV (Figure 3b) (see Supporting Information Section S10 for details), which is consistent with the results of STS. Furthermore, the photoluminescence spectrum (PL) for Fe_0.17_ZrSe_2_, as shown in Figure S7, exhibits the absence of peaks in the photon energy range of 0.41–0.60 eV from 77 to 300 K, consistent with an indirect band gap semiconductor (see Supporting Information Section S10 for details).

### Variable Temperature Magnetic Measurements

**Figure 4:**
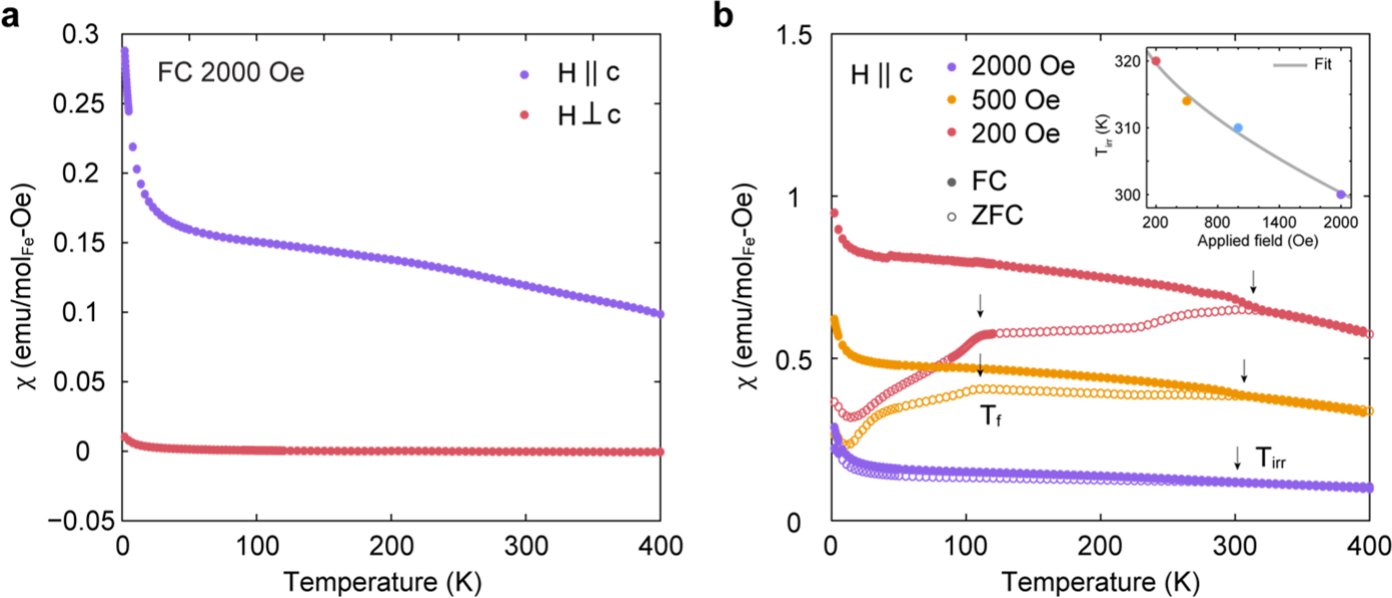
Temperature dependence of magnetic susceptibility of Fe_0.17_ZrSe_2_. (a) Temperature dependence of out-of-plane (purple dots) and in-plane (red dots) dc magnetic susceptibility of Fe_0.17_ZrSe_2_ single crystals measured with field cooling (*μ*_0_*H*_FC_ = 2000 Oe). (b) Temperature dependence of out-of-plane zero-field-cooled (ZFC) and field-cooled (FC) dc magnetic susceptibility for an Fe_0.17_ZrSe_2_ single crystal under applied magnetic fields ranging from 200 to 2000 Oe. The ZFC and FC data at a given field are represented by open and closed symbols, respectively. Inset shows the temperature of bifurcation, *T*_irr_, of ZFC and FC traces for different applied fields (solid dots) fit to the equation *T*_irr_(*H*) *= T*_irr_(0)(1 – *AH*^*n*^).

Figure 4a shows temperature-dependent magnetic susceptibility data for Fe_0.17_ZrSe_2_ acquired with a magnetic field of 2000 Oe applied along (purple) or perpendicular (red) to the *c*-axis of the crystal. These data show that the in-plane magnetic susceptibility for Fe_0.17_ZrSe_2_ is substantially weaker than the out-of-plane magnetic susceptibility, revealing strong magnetocrystalline anisotropy (MCA), consistent with the previous reports.^39^ This MCA may arise from the trigonally distorted pseudooctahedra of the majority of Fe^2+^ centers. We observe an ∼3.4% trigonal compression from the size of the Fe octahedral site extracted from SCXRD, which results in the qualitative *d*-orbital splitting depicted in Figure S8. The unevenly occupied *e*_*g*_ (*d*_*xy*_, *d*_*x^2^–y^2^*_) set of octahedral high-spin Fe^2+^ would result in unquenched orbital angular momentum and thus MCA.

As a function of temperature, the magnetization of Fe_0.17_ZrSe_2_ between 350 and 400 K follows the Curie–Weiss law with *χ = C*/(*T – θ*_CW_) (Figure S9), where *C* and θ_CW_ are the Curie constant and Curie–Weiss temperature, respectively. We find θ_CW_ = −223 K, consistent with the predominance of antiferromagnetic exchange interactions in the high temperature regime. *C* is determined to be 3.20 emu K/(mol_Fe_ Oe) and the calculated effective magnetic moment *μ*_eff_ of 5.06 is close to the theoretical value of 4.90 for high spin Fe^2+^ ions, assuming *g* = 2 and *S* = 2 (see the Supporting Information for details). Figure 4b depicts zero-field-cooled (ZFC) and field-cooled (FC) dc magnetic susceptibility under various applied fields ranging from 200 to 2000 Oe. These ZFC and FC curves bifurcate around 300 K, arising from irreversibility induced under different cooling protocols. Such bifurcation has been reported in spin-glass and superparamagnetic systems.^39,62−65^ To investigate the nature of this state, we determined the irreversible temperature *T*_irr_ at each applied field by identifying the bifurcation point of the ZFC and FC curves. In the low-field region, the *T*_irr_ can be described as a function of the applied magnetic field, *H*, by the following expression:
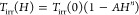
where *A* is a constant and *T*_irr_(0) is the limit of the irreversible temperature in the absence of a magnetic field. Theoretical models for spin-glass systems predict that in the limit of weak magnetic fields, the spin freezing temperature with weak irreversibility follows the Gabay–Toulouse (GT) line (*T*_irr_ ∝ *H*^2^), while the Almeida–Thouless (AT) line (*T*_irr_ ∝ *H*^2/3^) is followed by systems with strong irreversibility characteristics.^66,67^ The experimentally observed field dependence of *T*_irr_ along with the fit to the above equation are shown in the inset of Figure 4b. The value of *n* obtained from the fit is 0.63, which reveals that *T*_irr_ follows approximately *H*^2/3^ behavior that is consistent with the strong irreversibility of the glassy system.

In Figure 4b, we observe that with decreasing temperature below *T*_irr_, the ZFC curves display a plateau in the measured moment followed by a downturn, whereas the FC curves show continuously increasing moments. ZFC curves in applied fields of 200 and 500 Oe reveal a broad peak near 110 K, and the ZFC trace measured in a field of 500 Oe also shows a transition near 40 K. The upturns observed below 14 K are likely a Curie tail from a paramagnetic impurity. Ac heat capacity measurements of Fe_0.17_ZrSe_2_ in the absence of a magnetic field (Figure S11) show that no distinct thermodynamic phase transitions are present. This absence of a response in heat capacity measurements is consistent with glassy order.^68^ The magnetization measurements with applied field perpendicular to the *c*-axis of the crystal also show a bifurcation between the ZFC and FC traces (Figure S12), revealing that the spin glass behavior is also anisotropic.

### Relaxation of Magnetization for the Spin-Glass Phases of Fe_0.17_ZrSe_2_

**Figure 5:**
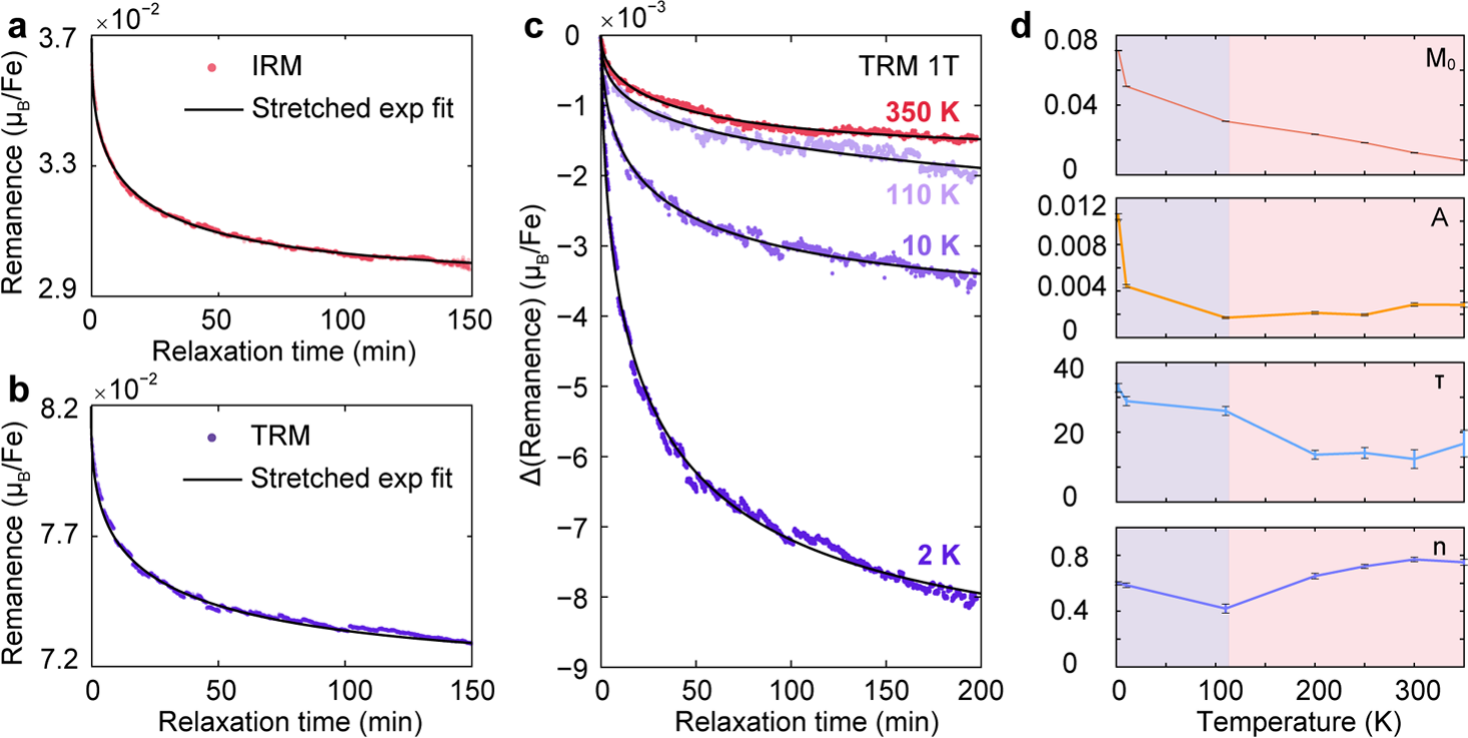
Slow magnetic relaxation behavior of Fe_0.17_ZrSe_2_. (a, b) IRM (red dots) and TRM (purple dots) data collected from the protocol described below with 1 h wait time, respectively, and their corresponding fits (black lines). The material was first fast-cooled from 400 to 60 K at 10 K/min and then slow-cooled from 60 to 2 K at 1 K/min under (a) zero field or (b) applied field of 1 T, and then held in applied field of 1 T for a designated wait time, t_w_, at 2 K. The field was then removed, and the IRM or TRM data were collected. The appearance of relaxation dynamics is correlated with the glassy state. Both IRM and TRM show similar dynamics, which indicates a common relaxation mechanism in both routines. (c) TRM measurements (dots) performed at various temperatures after the samples were field cooled in a field of 1 T and their corresponding fits (black lines). The remanences at *t* = 0 min for all of the TRM curves are normalized to 0 *μ*_B_/Fe. All of the fits in the figure were performed using a typical stretched exponential decay function *M*_R_(*t*) = *M*_0_ + *A* exp[−(*t*/τ)^1–*n*^]. (d) The extracted parameters from the fitting were plotted as a function of temperature. The fittings for the temperatures under 110 K are shaded with purple, whereas the fitting for the temperatures higher than 110 K are shaded with red.

A multivalley energy landscape arising from spin frustration emerges when cooling a spin glass through its freezing temperature.^62,64,69^ The nonergodicity of the glass system in turn engenders complex magnetic relaxation processes that are apparent in isothermal remanent magnetization (IRM) and thermoremanent magnetization (TRM) measurements as a slow relaxation of the magnetization over time (Figure 5). For the investigation of the slow relaxation dynamics of Fe_0.17_ZrSe_2_, IRM and TRM measurements were performed following the protocols outlined in Figures S13a and S13b, respectively. In short, IRM measurements were carried out by cooling samples down to the target temperature without application of a field, applying a field for a given wait time (*t*_*W*_), removing the field, and measuring the magnetization as a function of time. TRM measurements were carried out by cooling to the target temperature in the presence of a field, holding at this applied field for *t*_W_, removing the field, and then monitoring the change in magnetization as a function of time. See the Supporting Information for additional methodological details. Figure 5a, b shows that the magnetization relaxes in time upon removal of the applied field, a characteristic of glassy systems. Moreover, the relaxation behavior for both IRM and TRM were best fit using the typical stretched exponential decay function of
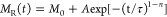
where *M*_0_ is the remanent magnetization at *t* = ∞ (that is, the intrinsic component of the moment), *A* is the peak beyond equilibrium values related to glassy component of the magnetization, τ is the characteristic average relaxation time, and *n* is the time stretch component. The obtained τ values at 2 K are around 17 and 24 min, and the *n* is found to be approximately 0.58 (Table 1). Both values fall in the typical range of glassy systems.^69,70^ Moreover, the relaxation times and time stretch components of the IRM and TRM measurements are comparable, indicating a common relaxation mechanism in both routines.

**Table 1:** Magnetization Relaxation Parameters from Isothermal Remanent Magnetization (IRM) and Thermoremanent Magnetization (TRM) Measurements at 2 K

	*M*_0_(μ_B_/Fe)	*A*(μ_B_/Fe)	τ (min)	*n*
TRM	0.0717	0.0104	23.7	0.590
IRM	0.0295	0.00745	16.6	0.571

To investigate the temperature-dependent dynamics of the glassy state, TRM measurements were conducted at selected temperatures, as shown in Figure 5c and Figure S14. The corresponding fitting parameters are presented in Figure 5d and Table S4. These measurements show that the slow relaxation behavior persists at room temperature. Notably, *A* and τ appear temperature dependent for temperatures between 2 and 110 K and both parameters decrease with increasing temperature (Figure 5d), as expected for a system trapped in metastable states separated by finite energetic barriers. Above 110 K, these parameters appear largely independent of temperature, even as *M*_0_ decreases steadily (yet producing a finite *M*_0_ value up to 350 K). The onset of the long, temperature-dependent τ values is consistent with the *T*_f_ value of 110 K, while the persistent slow relaxation above 110 K may be indicative of a disparate glassy state that is characterized by the bifurcation in the FC/ZFC traces and the *T*_irr_ value of Figure 4b. Furthermore, the decay curves in Figure S15 indicate that longer *t*_W_ values are associated with higher magnetization values, which is another characteristic feature of a glassy magnetic system.^62,69^

### Magnetic Hysteresis and Exchange Bias

**Figure 6:**
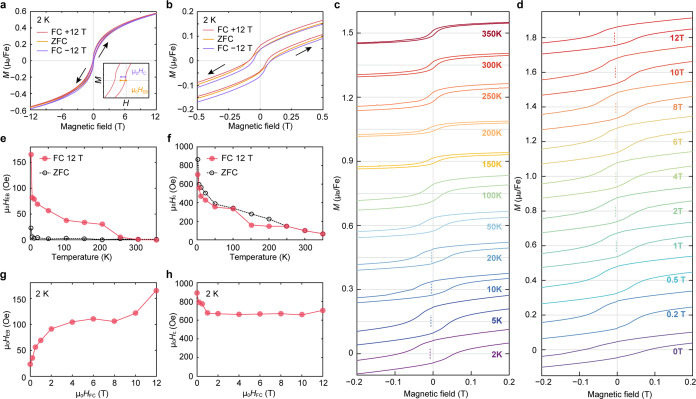
Exchange bias. (a) Magnetic hysteresis loops measured after the samples are cooled under 12 T (red curve), −12 T (purple curve), and zero field (orange curve) from 400 to 2 K. The inset in a demonstrates the definition of exchange bias field *μ*_0_*H*_EB_ (orange), and coercive field *μ*_0_*H*_C_ (purple). *μ*_0_*H*_EB_ was extracted from each loop by taking the average of the *x* intercepts, *μ*_0_*H*_EB_ = (*μ*_0_*H*_int1_ + *μ*_0_*H*_int2_)/2; *μ*_0_*H*_C_ was calculated from the half-width of the hysteresis loop at the average of the *y* intercepts. (b) Expanded view of the region between −0.5 and 0.5 T in a. The shifts of the hysteresis loops collected under applied fields are symmetrical relative to the zero-field loop. (c) Magnetization versus magnetic field measurements performed on Fe_0.17_ZrSe_2_ at various temperatures. Each loop is offset on the *y*-axis by 0.15 *μ*_B_/Fe. (d) Magnetization versus magnetic field measurements performed on Fe_0.17_ZrSe_2_ under various cooling fields. Each loop is offset on the *y*-axis by 0.2 *μ*_B_/Fe. (e, f) Temperature dependence of the extracted (e) exchange bias field *μ*_0_*H*_EB_ and (f) coercive fields *μ*_0_*H*_C_ after cooling in a 12 T (red trace) and 0 T (black dotted trace) from 400 K. (g, h) Cooling field dependence of (g) *μ*_0_*H*_EB_ and (h) *μ*_0_*H*_C_ after cooling from 400 to 2 K. All the hysteresis loops were collected with the magnetic field applied along the *c*-axis of the samples.

Measurements of magnetization as a function of applied field were performed at 2 K after zero-field cooling of the sample. As depicted in Figure 6a, b, the magnetization of the system exhibits a hysteresis loop with a coercive field, *μ*_0_*H*_c_, of 866 Oe. At the highest measured applied field of 12 T, the hysteresis loop does not fully saturate, and the moment is measured to be 0.6 μ_B_/Fe (substantially lower than the μ_eff_ of 5.06 measured from C–W fits (Figure S9). Variable-field magnetization data were also collected at 2 K after cooling the sample under applied fields of +12 and −12 T (Figure 6a, b), revealing hysteresis loops with distinct lateral shifts from near zero to ∓166 Oe, respectively, accompanied by a shift along the vertical axis. Upon cooling in a positive (negative) field, the loop exhibited a bias in the negative (positive) direction, which is a signature of exchange bias.^2,17^ The magnitude of the exchange bias field, *H*_EB_, was calculated as
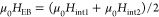
where *μ*_0_*H*_int1_ and *μ*_0_*H*_int2_ are the intercepts on horizontal axes. The magnitude of *μ*_0_*H*_c_ is defined as the half-width of the hysteresis loop at the average of the vertical intercepts, as depicted in the inset of Figure 6a.

Plots of *M*(*H*) over a range of temperatures are shown in Figure 6c and the associated temperature-dependent evolution of the exchange bias field *μ*_0_*H*_EB_ and the coercive field *μ*_0_*H*_c_ for *μ*_0_*H*_FC_ = 12 T (compared to the corresponding ZFC case) are presented in Figures 6e, f. The value of *μ*_0_*H*_EB_ decreases with increasing temperature, and a finite *μ*_0_*H*_EB_ persists up to at least 250 K. Likewise, *μ*_0_*H*_c_ decreases with increasing temperature, but the hysteresis loop is not completely closed at 350 K.

The exchange bias behavior at 2 K was also probed at different cooling fields, as shown in Figure 6d, g, h. Substantial increases in *μ*_0_*H*_EB_ are observed with increasing cooling fields between 0 to 2 T and 8 to 12 T, but *μ*_0_*H*_EB_ appears unchanged by increasing cooling fields from 2 to 8 T. The value of *μ*_0_*H*_c_ decreases slightly with increasing cooling field for *μ*_0_*H*_FC_ ≤ 1 T and remains effectively invariant with cooling field beyond 1 T.

## Discussion

### Crystal Field Stabilization Energy and Steric Effects on Occupation of Intercalated Fe

The vdW interfaces of 2*H* and 1*T* polytype TMDs create both octahedral and tetrahedral sites for intercalated ions (Figure 1). However, the experimentally determined crystal structures of Fe- intercalated TMDs compounds show that intercalants usually occupy the octahedral sites exclusively, except for Fe_*x*_ZrSe_2_.^37^ The preference for octahedral over tetrahedral sites in Fe-intercalated TMD compounds can be related to the increased coordination number of high-spin Fe^2+^ in octahedral complexes, as well as the negative octahedral site preference energy (OSPE) arising from the difference in crystal field stabilization energies (CFSE) between octahedral and tetrahedral complexes.^37,71,72^ The small OSPE in *d*^6^ complexes suggests that geometric effects could play a role in determining the stability of complexes. An analysis of Se–Se distances in structures of Fe-intercalated TMDs reported so far (Figure S19) shows how geometric effects may explain the coexistence of Fe^2+^ in tetrahedral and octahedral sites in Fe_*x*_ZrSe_2_. The Se–Se distances in the Fe octahedron of Fe_*x*_*M*Se_2_ are comparable to analogous Se–Se distances in Fe_7_Se_8_ (in which Fe is exclusively octahedrally coordinated), and the Fe tetrahedron in Fe_*x*_ZrSe_2_ is the closest in size to that found in FeSe (in which Fe is exclusively tetrahedrally coordinated) by comparison to other host lattice TMDs. This simple analysis might explain why the tetrahedral intercalant site in 1*T*-ZrSe_2_ may more readily accommodate Fe than the corresponding sites in other TMDs, providing a possible explanation for the distribution of Fe intercalants in both tetrahedral and octahedral sites, which are observed by SCXRD, HAADF-STEM, and EXAFS.

### Spin Glass Behavior

Structural and spectroscopic characterization (Figure 2) demonstrates that Fe_0.17_ZrSe_2_ is a crystallographic single-phase material with Fe^2+^ ions distributed in a disordered fashion between the layers of 1*T*–ZrSe_2_, and the material remains semiconducting (Figure 3). Multiple measurements of this material display signatures of spin glass behavior up to room temperature. Magnetic susceptibility data (Figure 4) display a bifurcation in FC and ZFC measurements at a temperature, *T*_irr_ (around 300 K), that depends on magnetic field—a phenomenon typically attributed to the freezing of moments.^73,74^ This freezing occurs when the crystalline anisotropy of small domains overcomes thermal fluctuations, which leads spins to freeze in random directions. In the low-field region, *T*_irr_ decreases with increasing applied magnetic field and the dependence of *T*_irr_ with field (Figure 4b inset) shows that the system follows the Almeida–Thouless (AT) line with *T*_irr_ ∝ H^2/3^. This trend is consistent with Fe_0.17_ZrSe_2_ representing a glassy system with strong irreversibility characteristics. A negative Curie–Weiss temperature (*θ*_CW_ = −223 K) in the apparently paramagnetic regime (above 350 K) is consistent with the predominance of antiferromagnetic exchange interactions in Fe_0.17_ZrSe_2_ crystals. Indeed, a downturn in magnetic susceptibility near 110 K in ZFC measurements is observed under both 200 and 500 Oe applied fields (Figure 4b), which could be suggestive of a weak AFM transition, though no predominant phase transition is observed as evinced by specific heat capacity measurements (Figure S11). While the ZFC feature at 110 K does indicate a change in magnetic phase, such change in phase could be attributed to an AFM phase coexisting with a spin-glass phase either in the high temperature regime or in the low temperature regime. It could also simply correspond to two different spin-glass phases, where the secondary low temperature phase is reentrant.^75^

Measurements of magnetization over time (Figures 5 and Figure S14) reveal slow spin relaxation dynamics in Fe_0.17_ZrSe_2_ crystals, which is another characteristic feature of glassy magnetic materials. Below 110 K, the magnetization relaxation is strongly temperature dependent, with time constants varying from 33 to 26 min between 2 and 110 K. However, from 110 to 350 K, the time constant of the system is largely invariant with temperature (*ca*. 14 min). These relaxation data suggest two regimes of glassy behavior, with the coexistence of a putative AFM-like phase with a phase transition temperature of about 110 K (Figure 4b) serving to further frustrate the spin glass relaxation and significantly increase relaxation times. Such complex magnetic phases, involving the potential coexistence of spin glass and antiferromagnetic phases, might arise from the structural disorder induced by the intercalated Fe atoms occupying both tetrahedral and octahedral sites embedded between 1*T*-ZrSe_2_ layers and the competing exchange interactions between magnetic sites. The coexistence of spin-glass and antiferromagnetic phases has been reported in other Fe intercalated TMDs systems Fe_0.30_NbS_2_ and Fe_0.14_NbSe_2_.^24,76^ However, the behavior of Fe_0.17_ZrSe_2_ contrasts favorably with that of these other compounds, where Fe atoms occupy only the octahedral interstitial sites in the van der Waals gap of the host lattice. Although Fe_0.30_NbS_2_ and Fe_0.14_NbSe_2_ also exhibit a glassy behavior, the associated spin-glass phases are observed at *T* < 40 K and *T* < 10 K, respectively, considerably lower temperatures than the room temperature glassy behavior of Fe_0.17_ZrSe_2_ observed here. This may again be attributed to the occupancy of Fe in both octahedral and tetrahedral sites that introduces a more global intercalant disorder that may in turn produce an elevated freezing temperature.

### Exchange Bias Effect

Measurements of magnetization as a function of the magnetic field (Figure 6) display magnetic hysteresis with a finite coercivity, *μ*_0_*H*_c_, a behavior that is commonly associated with ferromagnetic systems. However, it is important to note that the opening of hysteresis loops has also been reported in several spin glass materials.^23,25,77^ Furthermore, the hysteresis loop measured under ZFC at 2 K does not saturate up to 12 T and the small effective moment, which can arise from a frustrated, multidegenerate ground state of the system,^66,69^ is also consistent with spin glass behavior in Fe_0.17_ZrSe_2_. Figure 6 also shows that when cooled in an applied field Fe_0.17_ZrSe_2_ exhibits a robust exchange bias. Both *μ*_0_*H*_c_ and the exchange bias field, *μ*_0_*H*_EB_, decrease upon increasing temperature, but a finite *μ*_*0*_*H*_c_ is observed up to 350 K and the onset of exchange bias (between 250 and 300 K) coincides with *T*_irr_ around 300 K. Once onset, the magnitude of the exchange bias appears independent with decreasing temperature until around 100 K, below which a large increase in *μ*_0_*H*_EB_ is observed.

Again, these behaviors point to two magnetic regimes of this predominant spin glass, separated by the possible onset of a weak, coexisting antiferromagnetic phase at *T*_f_ = 110 K. Between 2 K and *T*_f_, increasing temperature results in thermal fluctuations of the magnetization of both antiferromagnetic and spin glass phases, weakening the coupling between the AFM and SG, which leads to a sharp decrease in both *μ*_0_*H*_EB_ and *μ*_0_*H*_c_.^78^ The coexistence of spin-glass with other magnetic phases has been found in multiple systems besides Fe intercalated TMDs,^79−81^ and the interaction between spin-glass and antiferromagnetic phases in those systems have been extensively studied using the random-field Ising model.^82^ In artificial bilayer heterostructures of antiferromagnetic and spin glass materials, the interfacial pinning effect of antiferromagnetic domains on spins at the surface of frozen glassy states has been invoked as the origin of an observed exchange bias effect and the interplay between antiferromagnetic order and random fields associated with spin disorder can have a significant impact on the occurrence of spin flips in applied magnetic fields.^83^ Since it is the interplay between two magnetic phases that is generally thought to be the key factor giving rise to exchange bias in metallic multilayers, it is reasonable to expect the coexistence of spin-glass and antiferromagnetic phases in single crystallographic phase materials will also exhibit exchange bias behaviors. Nevertheless, these data show that the exchange bias can be observed even when the overwhelmingly dominant component is the spin-glass.

The increase in *μ*_0_*H*_EB_ with the cooling field, which we observe in the low-temperature, putative SG/AFM regime of Fe_0.17_ZrSe_2_ (Figure 6g), has also been observed in conventional FM/AFM multilayer systems. This effect is explained by considering that the cooling field would act on the AFM component to give rise to an additional induced magnetization. Correspondingly, the magnetic domain size would increase, and both the exchange coupling intensity of the interfacial domains and the unidirectional anisotropy would be strengthened,^84,85^ resulting in a stronger pinning interaction between AFM and SG phases that enhances *μ*_0_*H*_EB_.^86,87^ Likewise, the slight decrease in *μ*_0_*H*_c_ with cooling field that we observe (Figure 6h) can be explained by considering that larger cooling fields would increase the polarization of the spin glass^87^ weakly ordering this phase and reducing the pinning within the SG domain, leading to the suppression of *μ*_0_*H*_c_.

To assess the potential applicability of the exchange bias phenomenon in Fe_0.17_ZrSe_2_, we contextualize this system within a framework of representative exchange bias systems, as summarized in Table S7. The observed exchange bias in Fe_0.17_ZrSe_2_, measuring 166 Oe at 2 K, aligns with values found in various canonical heterostructure systems.^2^ While certain systems can achieve larger exchange bias values, even up to 3 T,^24^ rendering them suitable for constructing robust biased permanent magnets, a smaller robust bias in the range of hundreds of Oe is more advantageous for spintronics applications, such as creating a platform for magnetic RAM devices or the electrical manipulation of magnetism, due to its enhanced energy efficiency.

## Conclusions

Combined compositional, structural, and spectroscopic characterization reveals that Fe_0.17_ZrSe_2_ crystals exhibit high crystallinity of the 1*T*-ZrSe_2_ host lattice, while Fe atoms occupy both tetrahedral and octahedral sites, distributed in a disordered fashion between TMD layers. Magnetometry measurements of Fe_0.17_ZrSe_2_ demonstrate magnetic irreversibility under zero-field cooling (ZFC), slow relaxation dynamics, the absence of saturation, and a small effective moment under high applied magnetic fields. These findings unveil a frustrated and multidegenerate ground state in Fe_0.17_ZrSe_2_ single crystals that persists up to room temperature. Interestingly, a robust exchange bias is observed below 250 K, and the coexistence of spin glass phase and a nominal antiferromagnetic phase at temperatures below 110 K appears to contribute to an even more pronounced exchange bias at low temperatures.

Our results highlight the potential of intercalated TMDs like Fe_0.17_ZrSe_2_ for spintronics technologies that can operate near room temperature. The spin-glass-derived exchange bias observed here persists much higher than that found in other intercalated TMDs like Fe_0.33±δ_NbS_2_ and Fe_0.14_NbSe_2_, which display this behavior only below 40 and 10 K, respectively. Importantly, the semiconducting nature of Fe_0.17_ZrSe_2_ raises the prospect of strongly modulating or switching these magnetic phases with an electrical bias or optical excitation. More generally, exploring exchange bias in systems like Fe_0.17_ZrSe_2_ offers valuable insights into the role of disorder and frustration in magnetic materials more widely, including prototypical metallic magnetic multilayers. This work also opens doors to new design strategies for magnetic film interfaces that leverage materials with inhomogeneous microscopic environments to induce frustration and support exchange bias.

## Supporting Information Available

Materials and methods, experimental procedures and setup for synthesis and characterization, characterization data, and additional discussion (PDF)
