# Supplementary material for: Near Room-Temperature Intrinsic Exchange Bias in an Fe Intercalated ZrSe2 Spin Glass
Source: J Am Chem Soc. 2023 Aug 30;145(36):20041–52. doi: 10.1021/jacs.3c06967 (PMC10510322; doi:10.1021/jacs.3c06967)
Supplement: Supplementary file 1 — ja3c06967_si_001.pdf [file ja3c06967_si_001.pdf]

Supporting Information for

Near room-temperature intrinsic exchange bias in an Fe intercalated $ZrSe_2$ spin glass

Zhizhi Kong[1], Corey J. Kaminsky[2], Catherine K. Groschner[1], Ryan A. Murphy[1], Yun Yu[1], Samra Husremović[1], Lilia S. Xie[1], Matthew P. Erodici[1], R. Soyoung Kim[3], Junko Yano[2], and D. Kwabena Bediako[1,3]*

[1] Department of Chemistry, University of California, Berkeley, California 94720, United States

[2]Molecular Biophysics and Integrated Bioimaging Division, Lawrence Berkeley National Laboratory, Berkeley, CA 94720, USA

[3]Chemical Sciences Division, Lawrence Berkeley National Laboratory, Berkeley, CA 94720, USA

**Correspondence to: bediako@berkeley.edu*

Table of Contents

S1 Chemicals…… *3*

S2 Chemical vapor transport growth of Fe_xZrSe_2…… *4*

S3 Scanning electron microscopy and energy dispersive x-ray spectroscopy…… *5*

S4 Transmission electron microscopy and selected area electron diffraction…… *6*

S5 Single crystal x-ray diffraction…… *8*

S6 Raman spectroscopy…… *10*

S7 X-ray absorption spectroscopy…… *11*

S7.1 X-ray absorption near edge structure…… *12*

S7.2 Extended x-ray absorption fine structure fitting…… *13*

S8 X-ray photoelectron spectroscopy…… *16*

S9 Scanning tunnelling spectroscopy…… *17*

S10 Photoluminescence spectroscopy and UV−vis−NIR diffuse reflectance spectroscopy…… *19*

S11 Magnetometry measurements…… *21*

S12 Magnetocrystalline anisotropy of $Fe_{0.17}ZrSe_2$…… *22*

S13 Curie-Weiss fit…… *23*

S14 Out-of-plane magnetization of $Fe_{0.17}ZrSe_2$…… *24*

S15 Heat capacity…… *25*

S16 In-plane magnetization of $Fe_{0.17}ZrSe_2$…… *27*

S17 Relaxation analysis…… *28*

S18 Variable temperature magnetization data as a function of field…… *31*

S19 Steric effect on the occupation of Fe in vdWs gap…… *35*

S20 The contextualization of representative exchange bias systems…… *38*

S21 References…… *39*

S1. Chemicals

Unless otherwise stated, all materials and reagents were used as received.

The following chemicals were used for the chemical vapor transport (CVT) growth of crystalline iron intercalated zirconium diselenide (Fe_xZrSe_2). Iron powder (spherical, <10 µm, purity >99.9%, metal basis), and selenium powder (200 mesh, purity 99.999%, metals basis) were purchased from Alfa Aesar (Ward Hill, MA. USA). Zirconium pellets (catalog # ZR39X88-10G, purity >99.9%) was purchased from R.D. Mathis Co. (Long Beach, CA, USA). Iodine (catalog # 229695-20G, purity >99.999%, trace metals basis) was purchased from Sigma Aldrich Co., LLC. (St. Louis, MO, USA). $ZrSe_2$ single crystals were purchased from HQ graphene (Groningen, The Netherlands). The as-grown Fe_xZrSe_2 flakes were washed using toluene in an Ar-filled glove box. Glassware was oven-dried at a temperature of 150 °C for ≥ 4 h, and allowed to cool in an evacuated glove box antechamber prior to use. Anhydrous toluene was stored over 3- or 4-Å molecular sieves prior to use.

S2. Chemical vapor transport growth of Fe_xZrSe_2

Fe_xZrSe_2 was synthesized following procedures reported previously.[5] A mixture of 70.9 mg (0.778 mmol) of zirconium pellet, 123.0 mg (1.557 mmol) selenium powder, 24 mg (0.429 mmol) Fe powder along with transport agent 40.2 mg (0.158 mmol) of I_2 was loaded into 30 cm long quartz ampoule in atmospheric conditions. The ampoule was then evacuated to 1×10^{-2} Torr. The contents of ampoule were then cooled using liquid nitrogen to minimize the vaporization of I_2. When the pressure dropped below 1×10^{-4} Torr, the ampoule was sealed, and placed in a 3-zone furnace. The temperature settings for hot zone and cold zone are shown in **Figure S1a.** The heating and cooling rate was around 55 °C/h. **Figure S1c** shows that high-quality hexagonal crystals with diameters of several millimeters were obtained. SEM-EDS measurements (**Figure S2**) were used to determine elemental composition as Section S1.2

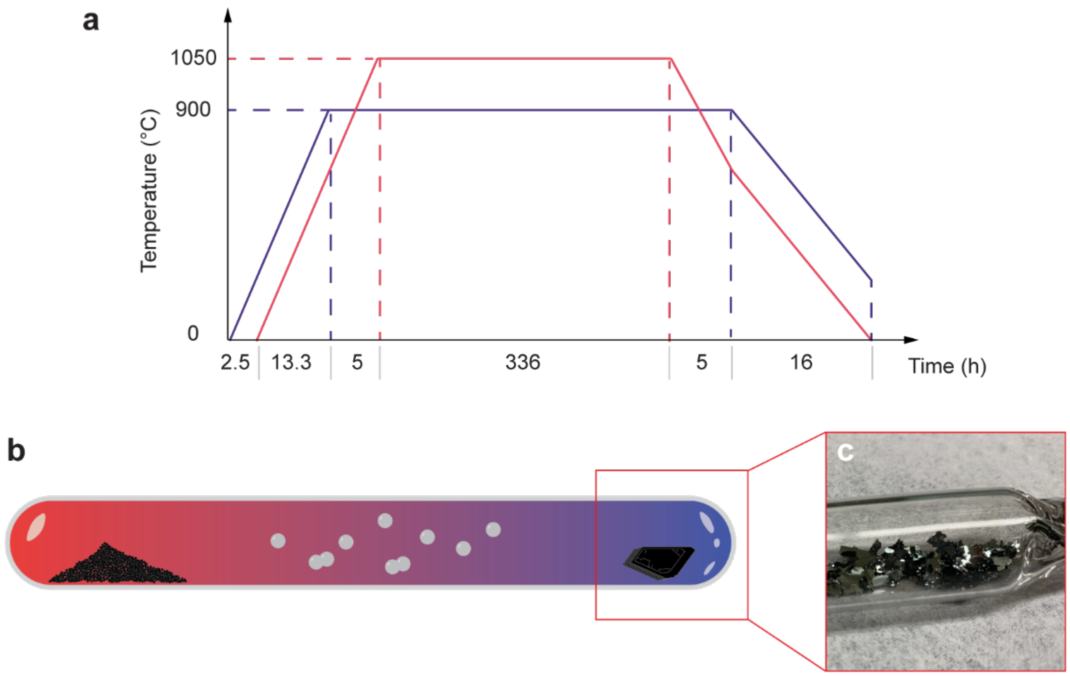

Figure S1. (a) Temperature settings of source zone (red line) and growth zone (purple line) for Fe_xZrSe_2 single crystal growth. (b) Schematic of a typical CVT experiment for bulk crystal growth of TMDs. Source materials, Zr pellets, Fe powder, and Se powder as well as transport agent I_2 are placed in one side of ampoule, which is evacuated, sealed, and positioned in a 3-zone furnace with a temperature gradient. (b) The image of as-synthesized iron intercalated $ZrSe_2$ crystals.

S3. Scanning electron microscopy and energy dispersive x-ray spectroscopy

Energy Dispersive X-ray Spectroscopy (EDS) measurements were performed on the crystal to extract the stoichiometry of iron intercalated between the $ZrSe_2$ layers. Scanning electron microscopy images were recorded on a Thermo Scientific Scios 2 DualBeam scanning electron microscope (SEM). Energy dispersive x-ray spectroscopy (EDS) of the Fe_xZrSe_2 crystals was performed with an Oxford Symmetry EDX detector using 20 keV accelerating voltage. Elemental compositions were determined by integrating under the characteristic spectrum peaks for each element using the AZtecLive Platform (Oxford Instruments NanoAnalysis).

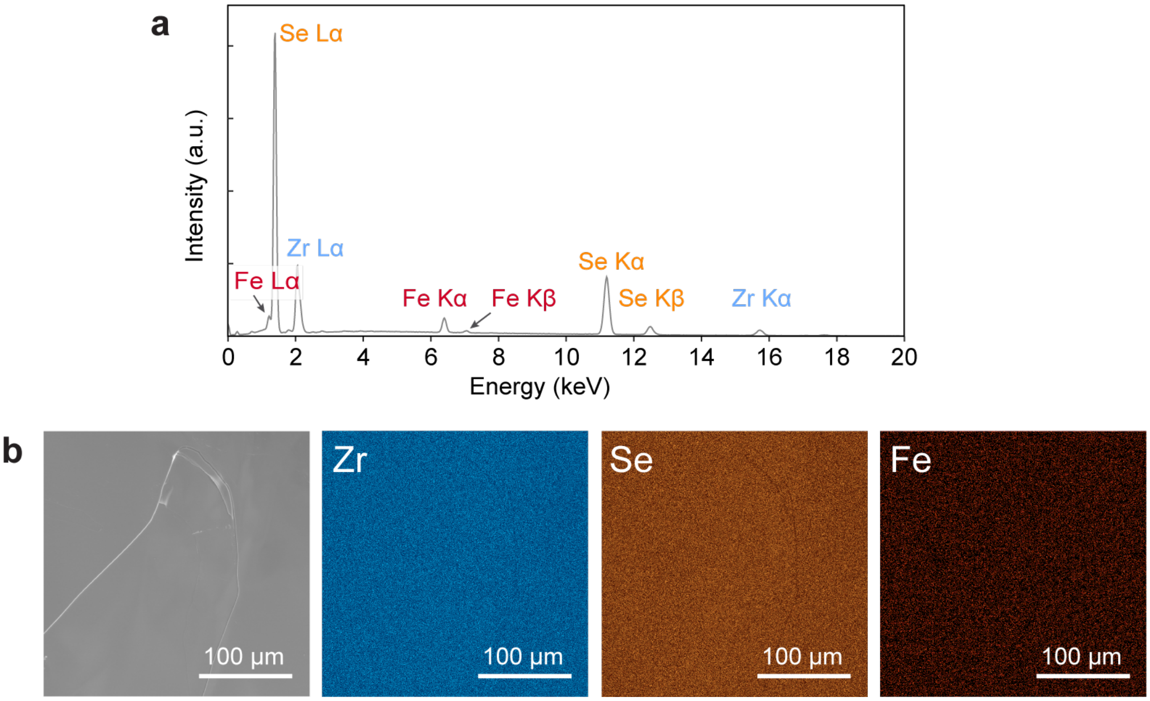

Figure S2. EDS measurement of the Fe-intercalated $ZrSe_2$ is presented. (a) The dispersion spectroscopy give an atomic ratio (Se:Zr:Fe) of 2.02:1:0.17. (b) SEM image and SEM-EDX mapping for elements of Zr, Se, and Fe for an as-grown $Fe_{0.17}ZrSe_2$ flake. The elemental maps reveal a uniform spatial distribution of Fe, Zr, and Se over a large area of approximately 90,000 μm^2.

S4. Transmission electron microscopy and selected area electron diffraction

TEM samples for imaging along the c-axis:

A single crystal of $Fe_{0.17}ZrSe_2$ was mechanically exfoliated with Blue Tape (Ultron Systems Inc.) and polydimethylsiloxane (PDMS) films (Gel-Pak) and transferred onto a NORCADA Location Tagged Micro-Porous TEM grid with 0.50 mm × 0.50 mm, 200 nm thick SiN_x membrane with 2-μm holes. Exfoliated flakes with thicknesses < 50 nm were identified using optical microscopy and atomic force microscopy. The TEM grid was cleaned in a vacuum annealer at 2.1×10^{-6} torr, 350 °C for 30 min immediately prior to stacking the sample onto the TEM grid.

Selected area electron diffraction (SAED) was performed with FEI TitanX microscope at the National Center for Electron Microscopy (NCEM) at the Molecular Foundry, Lawrence Berkeley National Laboratory. The SAED patterns were obtained at 60 kV applied voltage, collected along the $[10\bar{1}0]$ zone axis of the $Fe_{0.17}ZrSe_2$ flakes. The obtained patterns correspond to a 720 nm sample region, defined using a 40 μm diameter SAED aperture.

Cross-sectional samples for imaging along the *ab*-plane:

The cross-sectional TEM samples were prepared following procedures previously reported.[6] In brief, cross-sectional TEM samples were prepared with Thermo Scientific Scios 2 focused ion beam (FIB) scanning electron microscopy (FIB-SEM) systems. $Fe_{0.17}ZrSe_2$ flakes were exfoliated using Blue Tape and PDMS film onto a SiO_2/Si substrate. A 200 nm coating of Pt or C was deposited over the target $Fe_{0.17}ZrSe_2$ flake using an electron beam at 5 kV and 1.6 nA. This was followed by a deposition of 2.5 μm Pt or C layer using a gallium-ion beam at 30 kV and 0.1 nA. The initial bulk-out was performed with a 30 kV Ga beam and 3 nA current, while the bulk-out cleaning was performed with a 1 nA current. For the initial thinning, a 30 kV Ga beam was used, while the current was increasingly lowered as the sample became thinner. Initial thinning started with a 1 nA current, while the final current for the rough thinning was typically 0.3 nA. After the sample was approximately 150 nm thick, the FIB beam was switched to the 5 kV and 40 pA settings. The final polishing was performed at 2 kV and 40 pA to reduce the ion beam damage.

High-angle annular dark-field scanning transmission electron microscopy (HAADF-STEM) of cross-sectional TEM samples was performed on transmission electron aberration-corrected microscope (TEAM0.5) microscope at NCEM. The microscope was operated at 300 kV with a probe convergence angle of 30 mrad.

A single crystal of $Fe_{0.17}ZrSe_2$ was mechanically exfoliated with blue tape and transferred onto PDMS stamp/glass slide. Exfoliated flakes with thicknesses < 50 nm were identified using optical microscopy. The target flake was dropped onto a TEM grid with 200-nm-thick amorphous SiN_x membrane. Only diffraction peaks corresponding to the host lattice 1*T*-$ZrSe_2$ were observed while the diffraction peaks corresponding to ordered superlattices were not.

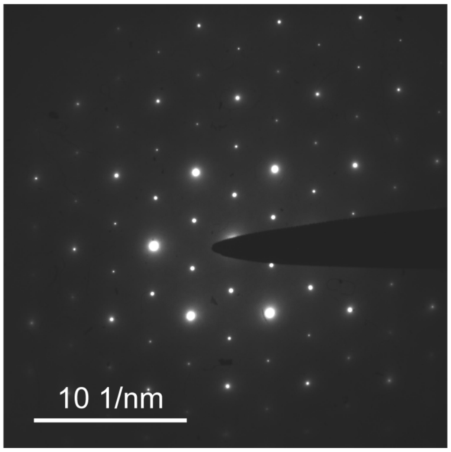

Figure S3. SAED pattern of a $Fe_{0.17}ZrSe_2$ flake.

S5. Single crystal x-ray diffraction

Single crystal x-ray diffraction (SCXRD) was obtained at UC Berkeley CHEXRAY crystallographic facility. SCXRD analysis was performed on a single crystal coated with Paratone-N oil and mounted on a MiTeGen MicroMount. Data were collected on a Rigaku XtaLab P200 (ChexTWOFACE) equipped with a MicroMax 007HF rotating anode and a Pilatus 200K hybrid pixel array detector using Mo Kα radiation (λ = 0.71073 Å). The temperature of the crystal was maintained at 100(2) K throughout collection. Data collection, refinement, and reduction were performed with CrysAlisPro (version 1.171.41.112a, Rigaku Corporation, Oxford, UK). A multi-scan absorption correction was applied using the SCALE3 ABSPACK scaling algorithm within CrysAlisPro. The structure was solved using direct methods with SHELXS[7,8] and refined with SHELXL (version 2014/07) ,[9,10] with refinement of F^2 on all data by full-matrix least squares, operated in the OLEX2[11] interface. All atoms were refined anisotropically. The 3D molecular structure figures were visualized with VESTA version 3.5.7 software,[12] while schematics were drawn in Autodesk 3ds Max and Adobe Illustrator version 27.3.1.

Table S1. Crystal data and structure refinement for $Fe_{0.17}ZrSe_2$

Empirical formula	Fe0.17Se2Zr	
Formula weight (g/mol)	258.63	
Temperature (K)	100	
Crystal system	Trigonal	
Space group	P-3m1	
Radiation	Mo Kα (λ = 0.71073)	
Unit cell dimensions	a = b = 3.7564(2) Å	$\alpha = \beta = 90°$
	c = 6.1078(4) Å	$\gamma = 120°$
Volume ($Å^{-3}$)	74.636(10)	
Z	1	
Density (calculated) (g/cm^3)	5.754	
Absorption coefficient (mm^{-1})	28.516	
F (000)	112.0	
Crystal size (mm^3)	0.075 × 0.05 × 0.003	
θ (°)	3.335 to 30.987	
Index ranges	$-5 \le h \le 5, -5 \le k \le 5, -8 \le l \le 8$	
Reflections collected	3773	
Independent reflections	114	
Completeness to θ_{full}	1.000	
Absorption correction	Semi-empirical from equivalents	
Refinement method	Full-matrix least-squares on F^2	
Data / restraints / parameters	114 / 6 / 11	
Goodness-of-fit on F^2	0.965	
Final R indexes [$I > 2\sigma(I)$]	R_1 = 0.0336, wR_2 = 0.0988	
Final R indexes [all data]	R_1 = 0.0353, wR_2 = 0.1021	
Largest diff. peak / hole (e $Å^{-3}$)	1.58 / −1.97	

S6. Raman spectroscopy

Raman spectra were acquired with a HORIBA LabRAM Evo Raman spectrometer using a 532 nm wavelength laser source. A 100× (NA = 0.9) objective (M Plan Achromat lens, Olympus Corporation) was used with a laser spot size of ~1 μm and a laser power of ~ 20 μW. Spectra were acquired with a grating of 600 grooves/mm, 1 s acquisition times, and 20 accumulations in ambient conditions (room temperature, 1 atm pressure). Higher laser powers (> 20 μW) and longer acquisition times (> 5 s) were found to lead to sample degradation.

S7. X-ray absorption spectroscopy

The XAS samples were prepared by sandwiching as-grown $Fe_{0.17}ZrSe_2$ crystals between two strips of KAPTON tape. XAS data were collected at the Stanford Synchrotron Radiation Lightsource (SSRL) beamline 4-3 at the Fe *K*-edge. Spectra were calibrated against an Fe foil. Samples were placed in a He filled chamber during data collection. No beam damage was observed during data collection.

Data were analyzed in the Demeter 0.9.26 suite of programs using Ifeffit 1.2.12. Spectra were imported to Athena for calibration, merging and spline fitting. The final spectra were imported into Artemis. Input files for path generation in Artemis were created using WebAtoms to convert CIF files into FEFF8 input files. The symmetry of the tetrahedral sites was not perfect, leading to slightly different length Fe–Se scattering paths. No significant difference was found between the choice of these paths. Both R_{eff} values for these paths are reported below with the unused path R_{eff} given in parentheses in the summary. **Table S2** is a summary of the paths used in the mode.

Table S2. Summary of the paths used in the mode

Type	R effective	Degeneracy
Fe-Se Octahedral	2.623	6
Fe-Fe Octahedral	3.756	6
Fe-Zr Octahedral	3.054	2
Fe-Se Tetrahedral	2.241 (alternative: 2.386)	4
Fe-Fe Tetrahedral	2.831	3
Fe-Zr Tetrahedral	3.050	3

S7.1. X-ray absorption near edge structure

Figure S4a shows the Fe K-edge XANES spectrum for $Fe_{0.17}ZrSe_2$. We attribute the pre-peak A (~7112 eV) to the local Fe tetrahedral ligand field that explicitly allows dipole transitions into 3*d* related states.[13] The intensity is low due to the low occupation of tetrahedral Fe. The feature B in the rising main absorption edge (the maximum point in the derivative absorption spectrum ~7118 eV) appears due to the 1s → 4p transition.[14] We assign the peak-like feature C (~7121 eV) to the 1s → 4p states admixed with the *d* states of the chalcogen atoms.[2] The D peak (~7125 eV) of 1s → 4p main transition is allowed by electric dipole matrix approximation.[15]

Figure S4b shows the comparison of Fe *K*-edge XANES spectra for $Fe_{0.17}ZrSe_2$ and the standard compound Fe^{2+} (FeO), Fe^{3+} ($LaSrFeO_4$), and Fe^{4+} ($SrFeO_{3-x}$)[1] and for the reported spectrum for FeSe.[2] The rising main absorption edge for $Fe_{0.17}ZrSe_2$ spectrum is observed close to the those of Fe^{2+} (FeSe) and Fe^{2+} (FeO), consistent with an oxidation state of +2, consistent with XPS result shown in the main text.

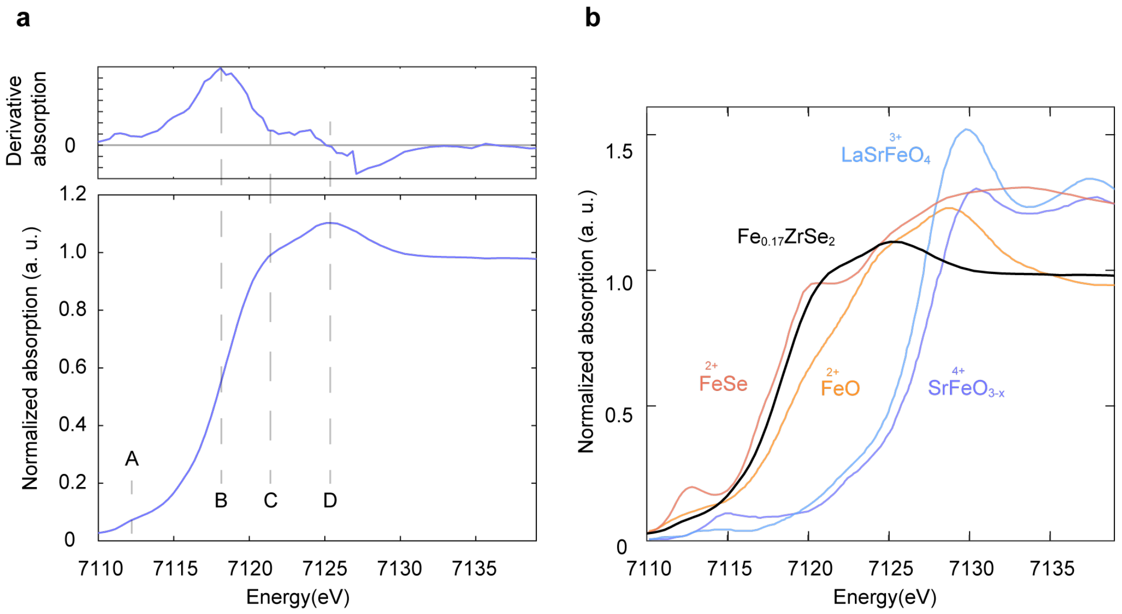

Figure S4. Fe K-edge X-ray absorption near edge structure (XANES) for $Fe_{0.17}ZrSe_2$. (a) Normalized Fe K-edge XANES spectrum for $Fe_{0.17}ZrSe_2$ and the corresponding first-order derivative absorption spectrum. (b) Fe K-edge XANES spectra for the standard compound Fe^{2+} (FeO), Fe^{3+} ($LaSrFeO_4$), and Fe^{4+} ($SrFeO^{3-}$) and for the reported FeSe, compared to that of $Fe_{0.17}ZrSe_2$. Fe^{2+} (FeO), Fe^{3+} ($LaSrFeO_4$), and Fe^{4+} ($SrFeO^{3-}$) spectra are adapted from ref. 1 FeSe spectrum is adapted from ref. 2

S7.2. Extended x-ray absorption fine structure fitting

EXAFS fits were performed against data in k-space with k, k^2 and k^3 weighted data for k between 3 and 12 $Å^{-1}$ with a Hanning window and in R-space for R between 1.6-4.2 Å. Each fit had 14.5 independent points with 9 variables. For all fits, the amplitude was set to 0.8, though qualitatively similar results were obtained with amplitude set to 0.7. The amplitude factor in the fits for the octahedral and tetrahedral paths were weighted by a factor x or $1-x$, respectively, where $x = 1$ corresponds to 100% octahedral sites and $x = 0$ corresponds to 100% tetrahedral sites. In a given fit, x was constant and a series of fits with the same parameters were performed where x was varied in steps of 0.05 or 0.1 from 0 to 1. Though the fit at 0.95 has the lowest overall value for the statistical assessments of the fit (i.e., reduced Red. χ^2 and R-factor) (**Figure S5**), this fit also contains an unreasonably large value for the change in path length for tetrahedral Fe–Fe scattering path, $\Delta R_{Td, Fe}$, and a negative value for the Debye-Waller analog for the tetrahedral Fe–Se path, $\sigma^2_{Td, Se}$. Thus, we identified $x = 0.9$ as the best fit. These fitting results are summarized in **Table S3.**

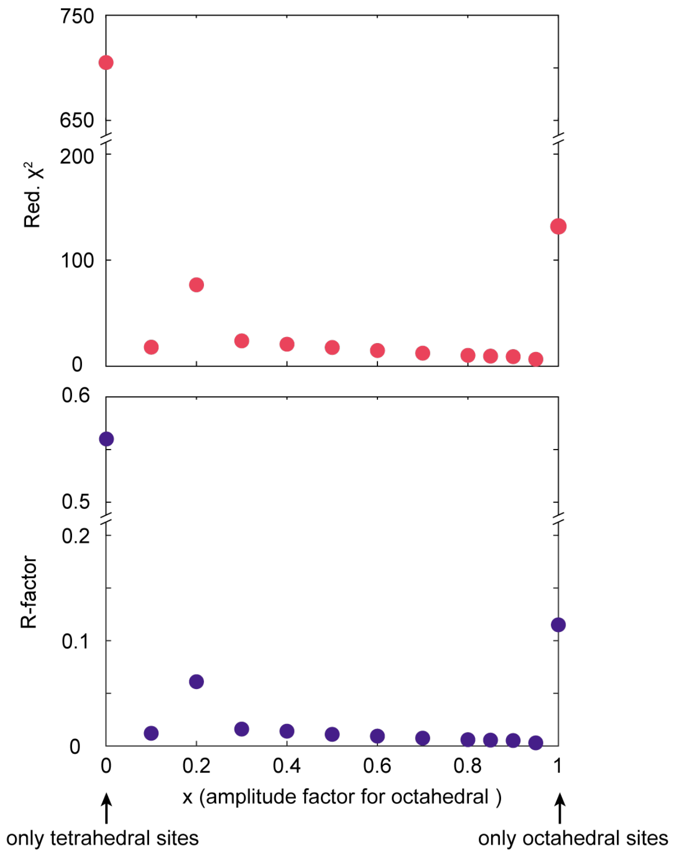

Figure S5. EXAFS fits against data in k-space with k, k^2 and k^3 weighted data. The statistical assessments of the fit, i.e., reduced Red. χ^2 and R-factor were plotted against the amplitude factor in the fits for the octahedral and tetrahedral paths. The octahedral or tetrahedral sites were weighted by a factor x or $1-x$.

Table S3. The fitting results of different ratios of octa-Fe to tetra-Fe

x	E^0	$\Delta R_{Oh, Se}$	$\sigma^2_{Oh, Se}$	$\Delta R_{Oh, Fe}$	$\sigma^2_{Oh, Fe}$	$\Delta R_{Td, Se}$	$\sigma^2_{Td, Se}$	$\Delta R_{Td, Fe}$	$\sigma^2_{Td, Fe}$	Red. χ^2	R-factor
1[a]	6.82	0.027	0.014	0.064	0.0378	0[b]	0.003[b]	0[b]	0.003[b]	131	0.115
0.95	4.0 ± 0.7	-0.003 ± 0.005	0.0146 ± 0.0003	0.04 ± 0.02	0.033 ± 0.002	0.091 ± 0.004	-0.0011 ± 0.0004	0.67 ± 0.02	0.001 ± 0.002	4.13	0.0029
0.9	4.8 ± 1.0	-0.007 ± 0.009	0.0120 ± 0.0007	0.04 ± 0.02	0.036 ± 0.004	0.11 ± 0.01	0.0016 ± 0.0007	0.03 ± 0.02	0.001 ± 0.002	6.64	0.0051
0.85	5.0 ± 1.1	-0.01 ± 0.01	0.0105 ± 0.0007	0.04 ± 0.02	0.035 ± 0.004	0.13 ± 0.01	0.0034 ± 0.0008	0.02 ± 0.02	0.002 ± 0.002	7.13	0.0055
0.8	5.5 ± 1.2	-0.00(7) ± 0.01	0.0093 ± 0.0007	0.04 ± 0.03	0.034 ± 0.004	0.14 ± 0.01	0.0049 ± 0.0009	0.02 ± 0.02	0.002 ± 0.002	7.88	0.0059
0.7	6.5 ± 1.4	-0.00(3) ± 0.01	0.0077 ± 0.0008	0.06 ± 0.03	0.032 ± 0.005	0.16 ± 0.02	0.007 ± 0.001	0.02 ± 0.02	0.004 ± 0.002	9.96	0.0074
0.6	7.5 ± 1.6	0.0(0) ± 0.01	0.0067 ± 0.0008	0.07 ± 0.04	0.029 ± 0.005	0.18 ± 0.02	0.010 ± 0.002	0.01 ± 0.02	0.005 ± 0.002	12.5	0.0093
0.5	8.5 ± 1.9	0.00(5) ± 0.01	0.0061 ± 0.0009	0.08 ± 0.04	0.027 ± 0.006	0.20 ± 0.03	0.013 ± 0.022	0.01 ± 0.02	0.007 ± 0.002	15.4	0.011
0.4	9.4 ± 2.1	0.01 ± 0.01	0.0055 ± 0.0009	0.10 ± 0.04	0.024 ± 0.006	0.21 ± 0.03	0.017 ± 0.003	0.00 ± 0.02	0.008 ± 0.003	18.5	0.014
0.3	10 ± 2	0.01 ± 0.1	0.0048 ± 0.0009	0.11 ± 0.05	0.019 ± 0.006	0.22 ± 0.04	0.022 ± 0.005	-0.00(5) ± 0.03	0.010 ± 0.003	21.7	0.016
0.2	4 ± 3	1.6 ± 0.1	0.01 ± 0.02	0.0(3) ± 0.1	.02 ± 0.01	0.09 ± 0.04	0.026 ± 0.006	-0.13 ± 0.02	0.0065 ± 0.0009	75.2	0.061

0.1	10 ± 2	-0.561 ± 0.009	0.004 ± 0.001	0.11 ± 0.04	0.007 ± 0.004	0.036 ± 0.008	0.014 ± 0.001	-0.11 ± 0.02	0.010 ± 0.001	15.7	0.012
0[a]	29	0[b]	0.003 [b]	0 [b]	0.003 [b]	0.085	0.0192	1.27	0.103	705	0.56

[a] Error bars of ± 0.000000 in model output for this row. [b] Initial guess value, parameter not fit under these conditions.

S8. X-ray photoelectron spectroscopy

X-ray photoelectron spectroscopy (XPS) measurements were performed using a Thermo Scientific K-Alpha^{+} (Al Kα radiation, hν = 1486.6 eV) (Thermo Fisher Scientific Inc) equipped with an electron flood gun. XPS data were analyzed using Thermo Scientific Avantage Data System software (version 5.9914), and a Smart background was applied prior to peak deconvolution and integration.

S9. Scanning tunnelling spectroscopy

Scanning tunneling spectroscopy (STS) measurements were performed on freshly cleaved bulk $Fe_{0.17}ZrSe_2$ crystals using a Park Systems NX10 STM module operated in ambient conditions. Pt–Ir tips were fabricated via electrochemical etching of 0.25 mm Pt–Ir wires in 1.5 M $CaCl_2$ solutions. The STS I–V curve was acquired by turning off the feedback loop, holding the tip a fixed distance above the surface, and sweeping the voltage from +0.8 to −0.8 V and then back to +0.8 V with duration of 1 s. The dI/dV trace was obtained by taking the derivative of the I–V curve.

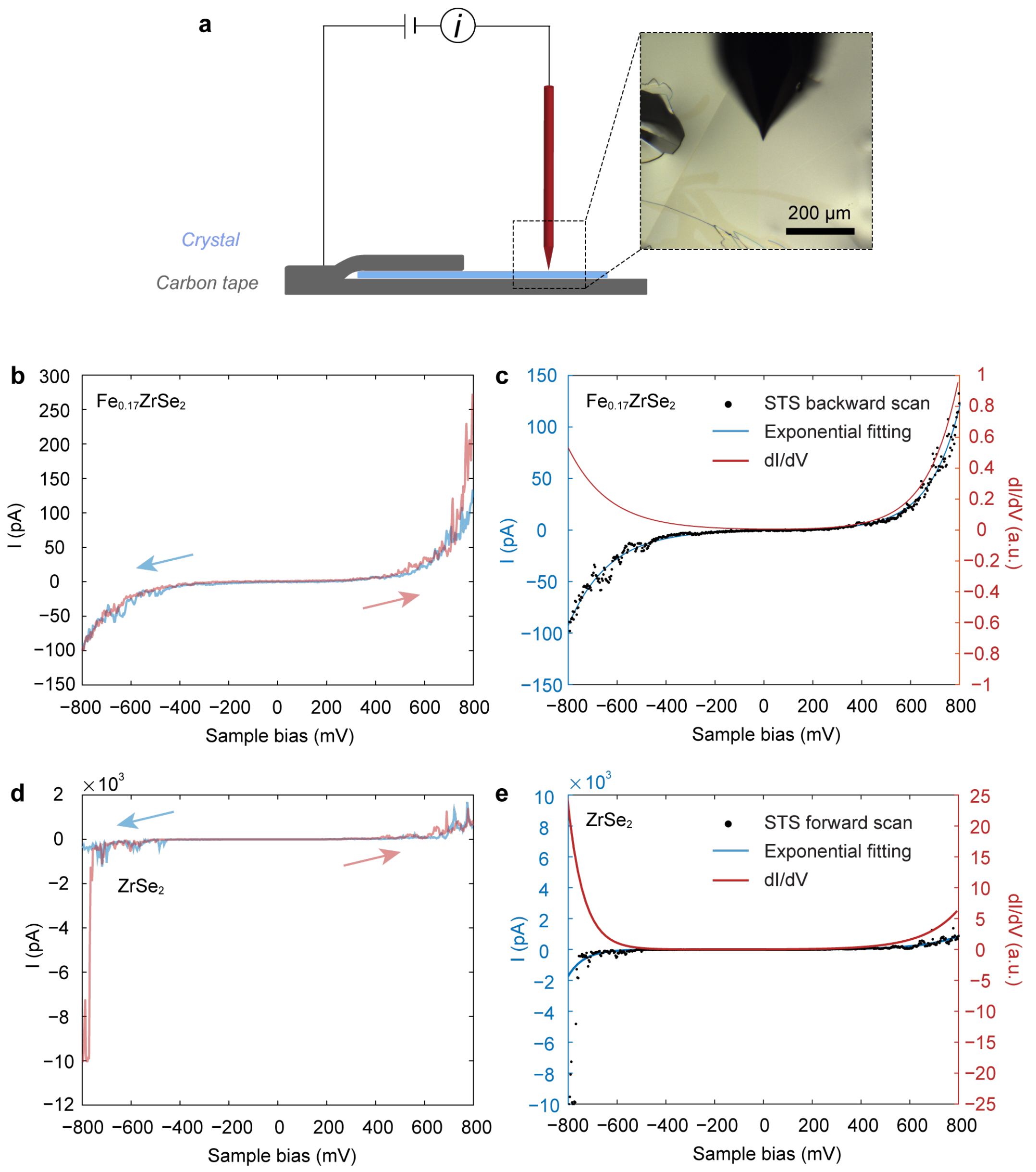

Figure S6. (a) Schematic of STS measurement setup. STS were performed on a freshly cleaved bulk sample in ambient conditions. The sample was mounted into the STS sample holder using carbon tape to attach the top of the sample to an STS probe. At a fixed tip–sample separation, the tunneling current was monitored while the bias voltage was swept from +0.8 V to –0.8 V (backward scan), and from –0.8 V to +0.8 V (forward scan). (b) The raw data of measured current of a $Fe_{0.17}ZrSe_2$ crystal with different applied sample bias. The backward scan is marked in a blue line and the forward scan is marked in a red line. (c) To mitigate the impact of raw data noise on the derivative of current versus sample bias, the raw data was fit with a two-term exponential model (blue line). The fitted data was used to obtain the dI/dV curve by taking the derivative of the current with respect to the sample bias (red line). (d) The raw data of measured current of a $ZrSe_2$ crystal with different applied sample bias and (e) the corresponding fitting and dI/dV curve. Data values at the extremes were excluded for the exponential fitting.

S10. Photoluminescence spectroscopy and UV−vis−NIR diffuse reflectance spectroscopy

Photoluminescence spectra (PL) were acquired with a HORIBA LabRAM Evo Raman spectrometer using a 532 nm wavelength laser source. For the ambient measurement (room temperature, 1 atm pressure), a 100× (NA = 0.9) objective (M Plan Achromat lens, Olympus Corporation) was used with a laser spot size of ~1 μm and a laser power of ~ 20 μW. Spectra were acquired with a grating of 600 grooves/mm, 3 s acquisition times, and 2 accumulations. Higher laser powers (> 20 μW) and longer acquisition times (> 5 s) were found to lead to sample degradation. For low temperature measurement, a 100× objective was used with a laser spot size of ~5 μm and a laser power of ~ 1 Mw. A sample was loaded in a Microscopy Cryostat (Model No.: CFM-1738-102, Cryo Industries of America, Inc. Manchester, NH, USA), which was attached to a Turbo Pumping station with Diagphram roughing pump (Model: HiCube 80 Eco, DN 63 ISO-K, MVP 015-4, Pfeiffer Vacuum Inc., Nashua, NH, USA) to maintain the pressure of 10^{-2} Pa during the measurement. Temperature is adjusted by a Temperature Controller (Model 325, Lake Shore Cryotronics, Inc., Westerville, OH, USA).

Photoluminescence spectra (PL) were collected on pristine $ZrSe_2$ crystals at room temperature in atmosphere (**Figure S7a**) and $Fe_{0.17}ZrSe_2$ in the temperature range from 77 to 300 K under vacuum (**Figure S7b**). No PL peak was observed for $ZrSe_2$, in line with $ZrSe_2$ being an indirect bandgap semiconductor, which is consistent with LAPW band calculations and experimental band structure obtained from ARPES.[16] Upon intercalation, **Figure 2** shows that the $ZrSe_2$ layers themselves do not undergo a phase change from. Accordingly, we suggest that a simple band filling and Fermi level renormalization process takes place upon intercalation. It seems reasonable to speculate therefore, that Fe intercalated $ZrSe_2$ persists as an indirect bandgap semiconductor. This conclusion is consistent with the PL measurements of $Fe_{0.17}ZrSe_2$ that display no peak from 77 to 300 K. Future ARPES measurements and/or theoretical calculations will be helpful in confirming the band structure of $Fe_{0.17}ZrSe_2$.

Diffuse reflectance spectroscopy measurements were performed on polycrystalline $Fe_{0.17}ZrSe_2$ samples, which were diluted in $BaSO_4$ and ground with a mortar and pestle to produce a homogenous powder in Ar-filled glove box. Diffuse reflectance UV-vis-NIR spectra were collected on a CARY 5000 spectrophotometer equipped with a Praying Mantis diffuse reflection accessory (Harrick Scientific Products, Inc.) and interfaced with Varian Win UV software.

The measured diffuse reflectance spectrum of $Fe_{0.17}ZrSe_2$ was transformed to the corresponding absorption spectrum by applying the Kubelka−Munk function:[17]

$$F(R_\infty) = \frac{K}{S} = \frac{(1 - R_\infty)^2}{2R_\infty}$$

where R_∞ is the diffuse reflectance, while K and S are the absorption and scattering coefficients, respectively.

The band-gap energy of semiconductors is usually determined by a Tauc plot,[18] which assumes that the energy-dependent absorption coefficient α could be interpreted by the following equation:

$$(\alpha \cdot h\nu)^{1/\gamma} = A\,(h\nu - E_g)$$

where h is the Planck constant, ν is the photon's frequency, A is a proportionality constant independent of the photon energy, and E_g is the band gap energy. The value of the γ factor is determined by the nature of the electron transition and is equal to 1/2 or 2 for direct or indirect transition band gaps, respectively.[19] According to photoluminescence spectrum (PL) for $Fe_{0.17}ZrSe_2$ in **Figure S7b**, no peak was observed in the energy range of 0.41 ~ 0.60 eV, which indicates that $Fe_{0.17}ZrSe_2$ is an indirect band gap semiconductor. Therefore, the $(F(R_\infty) \cdot E)^{1/2}$ was plotted against photon energy E in the main text. The *x*-intercept of the linear fit of the Tauc plot gives an estimate of the band gap energy, which is around 0.44 eV.

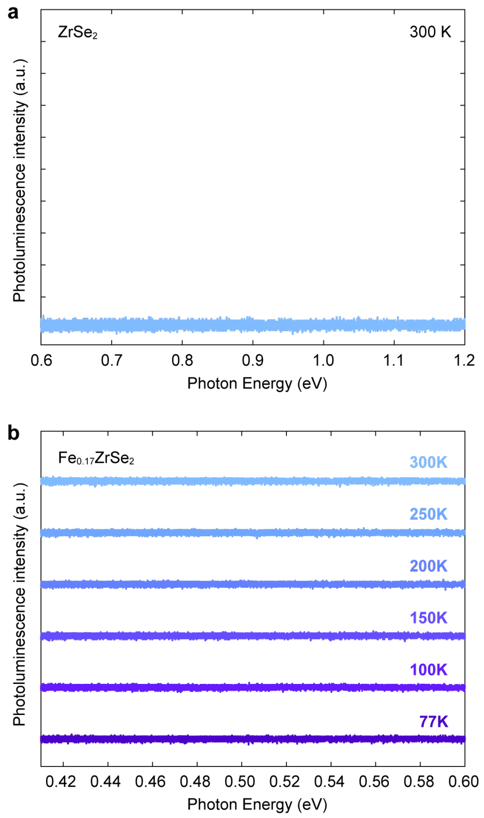

Figure S7. PL spectra for (a) pristine $ZrSe_2$ at room temperature subtracted by SiO_2/Si background. (b) $Fe_{0.17}ZrSe_2$ at temperature ranging from 77 to 300 K after subtraction of SiO_2/Si background.

S11. Magnetometry measurements

DC magnetization measurements as a function of temperature and applied field were carried out in a Quantum Design Physical Property Measurement System (PPMS) Dynacool equipped with a 12 T superconducting magnet using the Vibrating Sample Magnetometer (VSM) option, with a detection limit of 10^{-6} emu. For out-of-plane measurements, 7.89 mg of $Fe_{0.17}ZrSe_2$ flakes were stacked into polypropylene VSM powder sample holder, which was snapped into the brass half-tube. For in-plane measurements, 2.40 mg of $Fe_{0.17}ZrSe_2$ flakes were stacked onto MPMS 3 Quartz Paddle Sample Holder (C130A) and secured using Kapton tape.

S12. Magnetocrystalline anisotropy of $Fe_{0.17}ZrSe_2$

The fractional trigonal distortion is defined as $(a_2–a_1)/a_1$. For the regular octahedron, the fractional trigonal distortion is zero; for the trigonally elongated/compressed Fe environment, the fractional trigonal distortion is greater/smaller than zero. Values of a_1 = 3.253 Å and a_2 = 3.144 Å for $Fe_{0.17}ZrSe_2$ were exacted from the SCXRD solved structure. The fractional trigonal distortion is –3.35%, indicating that oct-Fe atoms in $Fe_{0.17}ZrSe_2$ locate in trigonally compressed distorted pseudo-octahedral coordination environment. The trigonally distorted pseudo-octahedron coordination environment gives rise to a qualitative d-orbital splitting diagram of e_g $(d_{xy}, d_{x^2-y^2})$, A_{1g} (d_{z^2}), and e_g (d_{xz}, d_{yz}).[3] XPS showed the oxidization state of Fe is +2. The weak crystal field results in a high-spin d^6 electron configuration for Fe^{2+} (S = 2). An unevenly occupied e_g set of Fe^{2+} leads to an unquenched orbital angular momentum and large spin orbit coupling.[3]

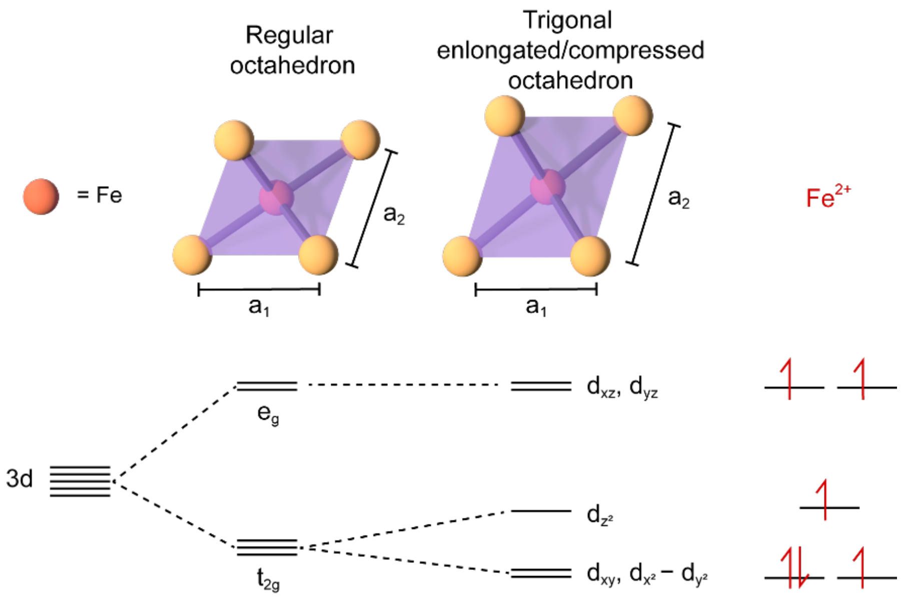

Figure S8. Schematic of perfect octahedral (left) and trigonally distorted (right) octahedral Fe environments, as seen along the [$2\bar{1}\bar{1}0$]] zone axis of the $Fe_{0.17}ZrSe_2$ lattice. Qualitative d-orbital splitting diagrams for intercalant high-spin Fe^{2+} in a trigonally distorted pseudo-octahedral coordination environment. Schematic adapted from ref. 3

S13. Curie–Weiss fit

Curie–Weiss fits were performed on the susceptibility data for $Fe_{0.17}ZrSe_2$ in the temperature range of 350 – 400 K. (**Figure S9**). The following functions were used to calculated effective moment μ_{eff}:

$$\chi = C/(T - \theta_{CW})$$

$$\mu_{eff} = \sqrt{8C}$$

where C is the Curie constant and θ_{CW} is the Curie-Weiss temperature. The extracted C, θ_{CW}, and μ_{eff} are shown below in **Figure S9**.

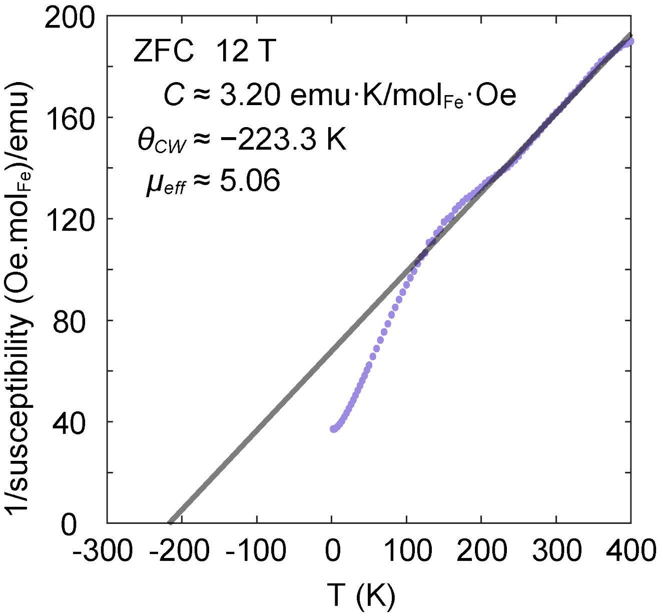

Figure S9. Inverse susceptibility as a function of temperature for $Fe_{0.17}ZrSe_2$ (purple dots). The data above 350 K was fitted to the Curie–Weiss model $\chi = C/(T - \theta_{CW})$(black line).

S14. Out-of-plane magnetization of $Fe_{0.17}ZrSe_2$

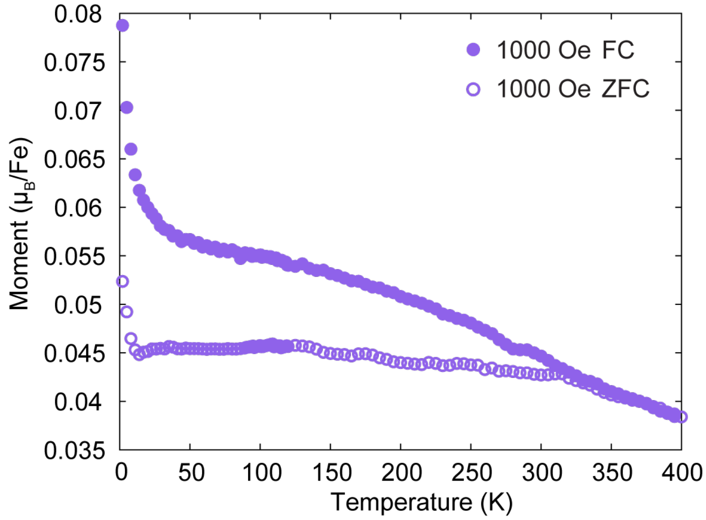

Figure S10. Temperature dependence out-of-plane zero-field-cooled (ZFC) (open dots) and field-cooled (FC) (filled dots) DC magnetization for $Fe_{0.17}ZrSe_2$ under applied magnetic fields 1000 Oe.

S15. Heat capacity

Heat capacity measurements as a function of temperature were carried out in a PPMS Dynacool using the Heat Capacity Option. 3.24 mg of $Fe_{0.17}ZrSe_2$ crystals were stacked together and compressed into one pellet and mounted onto heat capacity sample puck. The ac heat capacity measurement as a function of temperature was performed on this $Fe_{0.17}ZrSe_2$ pellet in the absence of an external magnetic field (**Figure S11a**). The specific-heat curve of $Fe_{0.17}ZrSe_2$ displays a broad transition but no sharp features, consistent with glassy order. The freezing of spins in different directions and the presence of frustration result in a distribution of energy levels. As a result, the heat capacity shows a broad maximum rather than a sharp peak.[4]

Further, the absence of sharp transitions in the specific heat capacity vs. temperature trace points to the homogeneity of the samples. The magnetometry data points to the coexistence of AFM and spin glass phases in the $Fe_{0.17}ZrSe_2$ below 110 K. If the two phases were segregated in discrete volumes throughout the sample, the specific-heat measurement would show a peak associated with the AFM transition overlaid with a broad glassy background. Therefore, the absence of the sharp transition reveals that there is no phase segregation in the sample.

A key characteristic indicating the presence of a spin glass state in the heat capacity measurement is its linear behavior of specific heat at low temperatures. A plot of the specific heat C_p as a function of T and the corresponding linear fitting for $Fe_{0.17}ZrSe_2$ at low temperature (< 3.5 K) are presented in the **Figure S11b**. A positive curvature takes place to force C_p to zero as temperature approaches 0 K as a consequence of the third law of thermodynamics. The linear temperature dependence of the specific heat in low temperature limit can be understood by the two-level tunnelling model, which is the simplified model from the rough energy landscape and macroscopic degeneracy of ground states in a spin glass. A sketch of a two-level energy diagram is shown in **Figure S11c**. There are two nearly similar energy minima separated with a tunnelling barrier (adapted from ref. 4). Assuming there is constant density of states for the two-level excitations. The quantum-mechanical tunnelling through the barrier between the two levels can result in a small rearrangement of some spins, which is temperature independent. Therefore, a linear proportionality of specific heat at low T naturally arises for the randomly frozen spin-glass.[4]

Figure S11d reveals a plot of the specific heat per unit temperature (C_p/T) as a function of T^2 for $Fe_{0.17}ZrSe_2$ at low temperature (< 10 K), which can be well expressed by using the following equation:[20-21]

$$C_P\ (\mathrm{T}) = \gamma T + \beta T^3 + \delta T^5$$

where γT describes the electronic contribution to the heat capacity and γ is the electron specific heat coefficient (Sommerfeld constant), βT^3 is the phonon contribution and β stands for phonon specific heat

coefficient, δT^5 and reflects the deviation term. As a result, the fitted values of parameters γ, β, and δ are 14.7(4) mJ/mol K^2, 0.55(3) mJ/mol K^4, and 1.2(3) × 10^{-3} mJ/mol K^6, respectively.

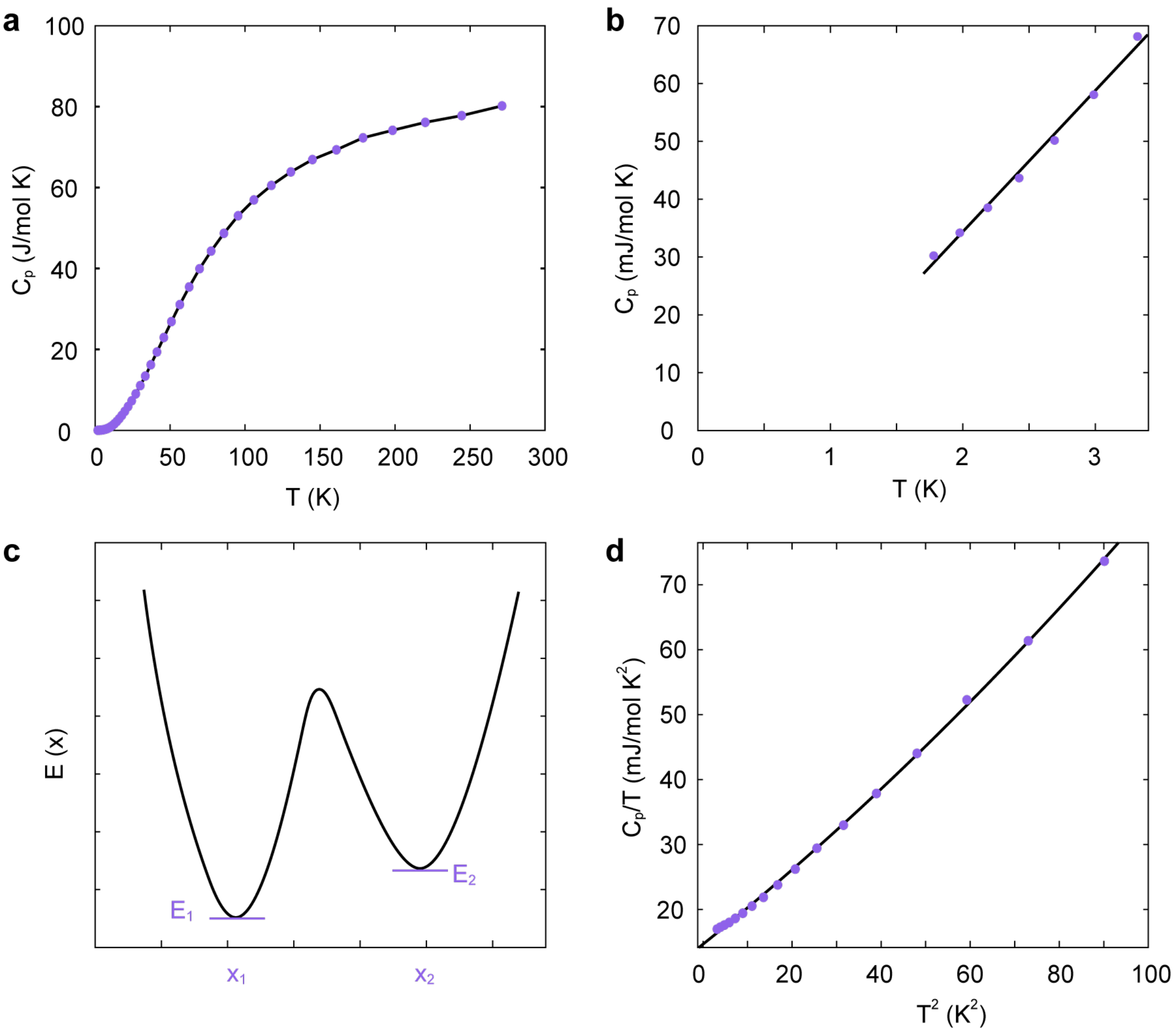

Figure S11. (a) Specific heat capacity (C_p) for $Fe_{0.17}ZrSe_2$ as a function of temperature (purple dots). Smoothed black line is a guide for the eyes. (b) Low temperature region of (a) (purple dots). The black line is the linear fitting curve. (c) A sketch of a two-level energy diagram (adapted from ref. 4). (d) Specific heat capacity divided by temperature (C_p/T) of $Fe_{0.17}ZrSe_2$ as a function of temperature squared (T^2) (purple dots). The black line is the curve of data fitted to C_P (T) $= \gamma T + \beta T^3 + \delta T^5$.

S16. In-plane magnetization of $Fe_{0.17}ZrSe_2$

The in-plane magnetization of the $Fe_{0.17}ZrSe_2$ crystals was measured as a function of temperature with the applied magnetic field parallel to the c-axis. The bifurcation between ZFC and FC curves reveals an anisotropic spin glass behavior along ab-plane, which has been reported before.[22] In Section S8, we discussed the magnetocrystalline anisotropy behavior along the *c*-axis in the $Fe_{0.17}ZrSe_2$ crystal, which might arise from the unquenched angular momentum of pseudo-octahedral Fe^{2+} coordination environment. However, it is worth noting that the *c*-axis cannot be exclusively considered as the easy axis, as spin glass behavior is also observed for fields perpendicular to the *c*-axis, although to a lesser extent. Therefore, the Fe_xZrSe_2 spin-glass system cannot be described according to a pure Ising model. Generally, the spin–orbit coupling effects in tetrahedral Fe^{2+} complexes are weaker compared to octahedral coordination environments. However, due to the disordered distribution of Fe at the octahedral and tetrahedral sites, a random distribution of spin directions, potentially canted with respect to the c-axis, is expected to emerge. Therefore, the Ising character of the octahedral Fe^{2+} spins may be significantly altered, and the anisotropic spin glass behavior may arise.[22]

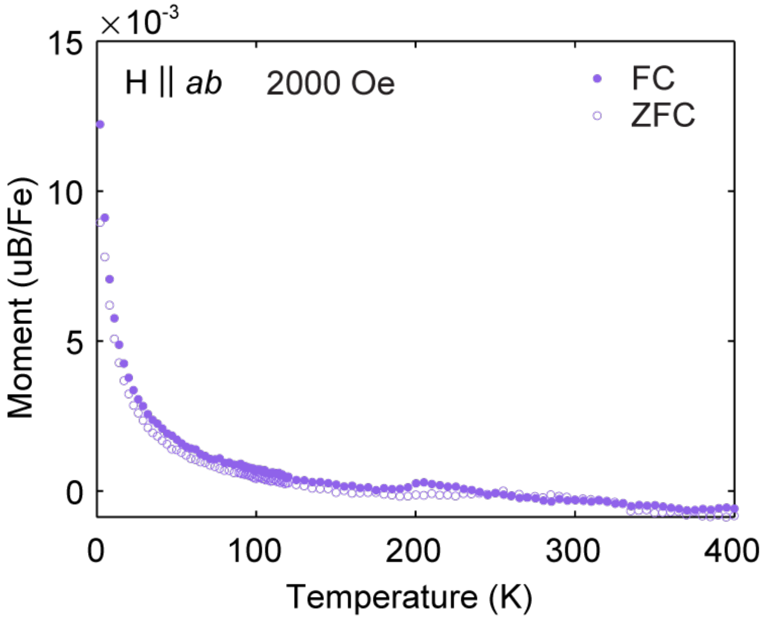

Figure S12. Temperature dependence in-plane zero-field-cooled (ZFC) and field-cooled (FC) DC magnetization for $Fe_{0.17}ZrSe_2$ under applied magnetic fields 2000 Oe.

S17. Relaxation analysis

Both isothermal remanent magnetization (IRM) and thermoremanent magnetization (TRM) measurements were performed on $Fe_{0.17}ZrSe_2$ following the protocols outlined in **Figures S13a** and **S13b**, respectively. TRM measurements were conducted in the Quantum Design PPMS with VSM option using the following protocol: (a) warm the sample to 400 K in zero magnetic field, (b) apply the field along crystallographic c-axis to 1 T, (c) fast cool the sample to 60 K above the target temperature at 10 K/min, (d) slow cool to the target temperature at 1 K/min, (e) hold the sample in 1 T field for wait time t_W one hour, and (f) set the field to 0 T and measuring the remanent magnetization in the sample over set time.

IRM measurements were conducted with same (a), (c)–(f) steps. For step (b), apply zero field along crystallographic c-axis.

The relaxation measurements for both IRM and TRM were best fit using the $M_R(t) = M_0 + A\exp[-(t/\tau)^{1-n}]$, where M_0 is remanence, irreversible part of the change in magnetization, A is peak beyond equilibrium values related to glassy component of the magnetization, τ is the characteristic relaxation time, and n is the time stretch component.

TRM measurements were conducted at different temperatures to study the effect of temperature on slow dynamics of $Fe_{0.17}ZrSe_2$ crystals. **Figure S14a** shows that the remanence decreases with increasing temperature, which was consistent with FC data. **Figure S14b and Table S4** reveals that in the range of 2 ~ 110 K, the parameters M_0, A, τ, and n changed drastically with increasing temperature. τ and n decreased with increasing temperatures, which suggests that energy barriers, which trap the system in its metastable state, are reduced at higher temperatures. When the temperature is higher than 110 K, the parameter M_0 still decreased with increasing temperature, indicating the polarization of spins in the $Fe_{0.17}ZrSe_2$ crystals is weaker at higher temperature. However, the $Fe_{0.17}ZrSe_2$ still exhibits slow relaxation behavior for T > 110 K and parameters A, τ, and n did not show obvious changes with temperature, consistent with the existence of a glassy phase at high temperature. We note that the fitting equation is not useful when T is higher than the freezing temperature, and relaxation curves at high temperature are noisy, making it challenging to extract precise relaxation parameters. Nevertheless, these fits provide some general characterization of the magnetic relaxation at high temperatures.

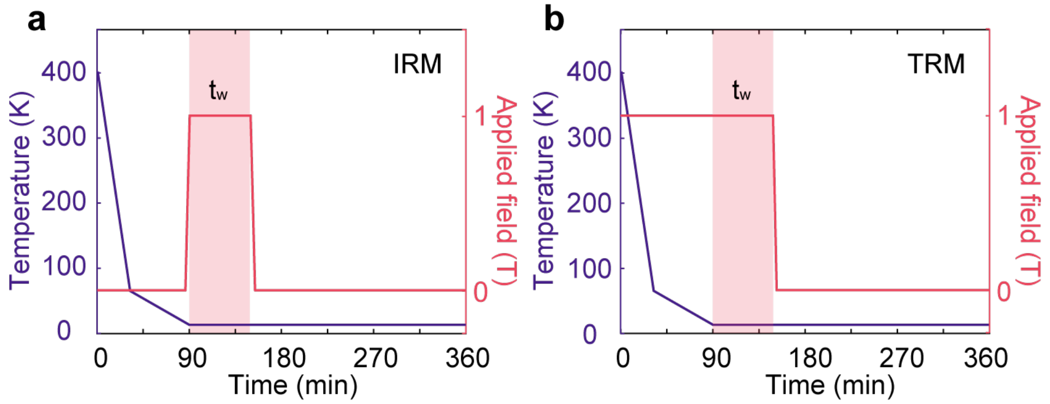

Figure S13. Illustration of isothermal remanent magnetization (IRM) measurements (a) and thermoremanent magnetization (TRM) measurements (b). The material was first fast-cooled from 400 K to 60 K by 10 K/min and then slow-cooled from 60 K to 2 K by 1 K/min under a 1 T applied field (a) or under zero field (b) and held in applied field 1 T for a designated wait time, t_W, at 2 K, the field was then removed and the TRM or IRM data were collected.

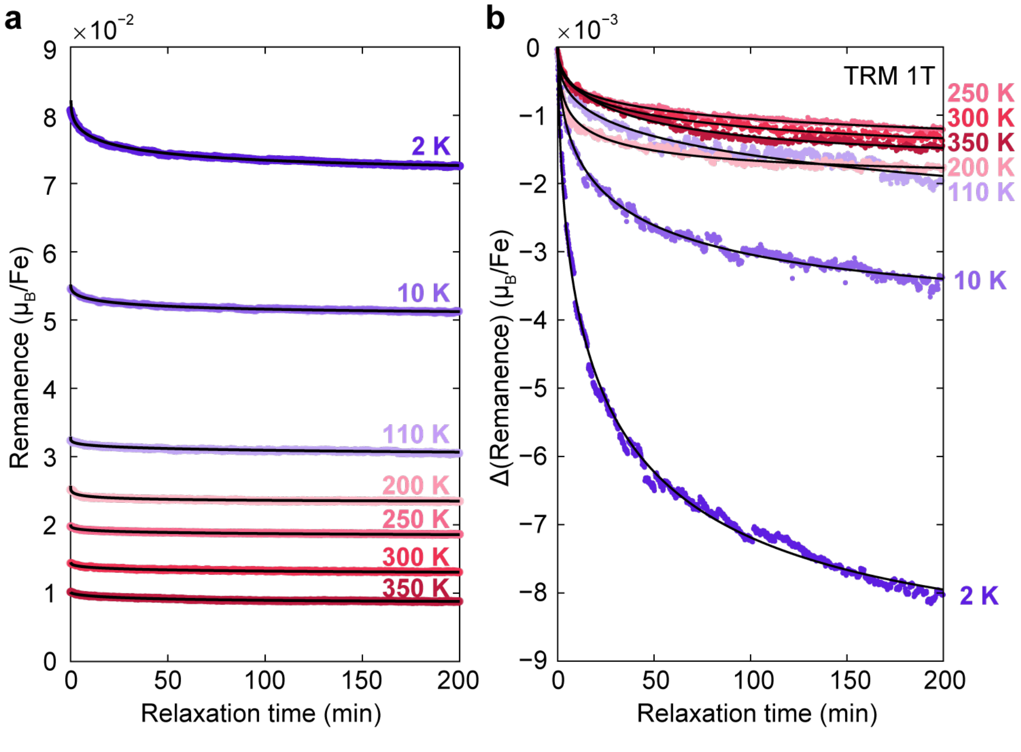

Figure S14. (a) The raw TRM curves at different temperature were obtained by FC in a 1 T magnetic field to the target temperature, waiting 60 min, removing this field, and finally measuring the variation in magnetization over 200 min. The TRM data plotted here are after field removal. (b) Change in TRM in (a) determined by subtracting the remanence at $t = 0$ min for all the TRM data (colored dots) and their corresponding fits (black lines).

Table S4. Temperature dependence of TRM relaxation fitting results

$$M_R(t) = M_0 + A \exp[-(t/\tau)^{1-n}]$$

Temp (K)	M_0 (μ_B/Fe)	A (μ_B/Fe)	τ (min)	n
2	0.07127	0.0104	32.74	0.5997
10	0.05078	0.004411	28.9	0.5866
110	0.03072	0.001706	26.1	0.4184
200	0.02328	0.002122	13.55	0.6517
250	0.01843	0.001949	14.06	0.7214
300	0.01271	0.002835	12.31	0.7707
350	0.008366	0.002811	16.73	0.7509

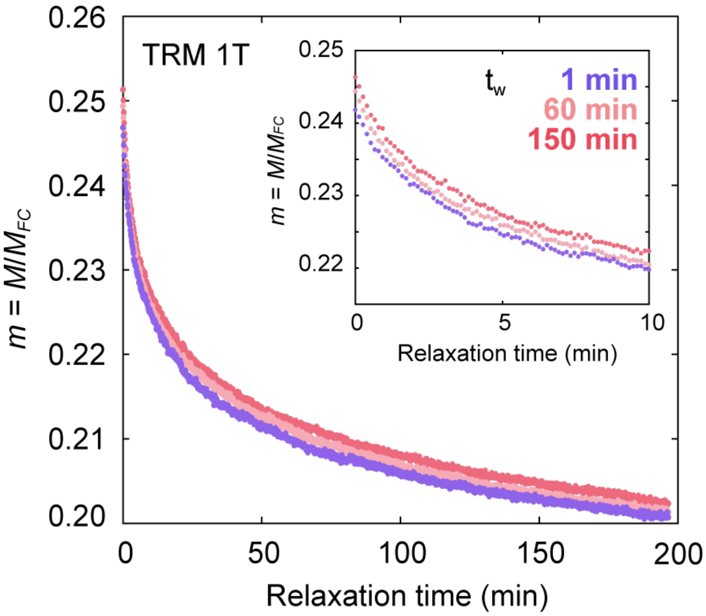

Figure S15. TRM data with different wait times. Inset: zoom in on the relaxation time ranging from 0 to 10 min. Magnetization increases with longer wait times, indicating the magnetic aging characteristic of spin glasses.

S18. Variable temperature magnetization data as a function of field

The temperature-dependent magnetization versus applied magnetic field scans were performed in the range of −12 T to 12 T. Zoomed in plots of FC magnetization sweep in the field range of −0.2 T to 0.2 T are presented in the main text. The side-by-side comparison of ZFC and FC plots are shown below, with zoomed-in plots in the range of −0.2 T to 0.2 T (**Figure S16**) and full range plots in the range of −12 T to 12 T (**Figure S17**).

Figure S18 shows the cooling field-dependent magnetization versus field scans performed at 2 K. The zoom-in plots of magnetization sweep in the field range of −0.2 T to 0.2 T were presented in the main text. The sample was field cooled down from 400 K to 2 K under the applied field and then the applied field was set from the cooling field to 12 T. All loops were taken from +12 T to −12 T and back to +12 T.

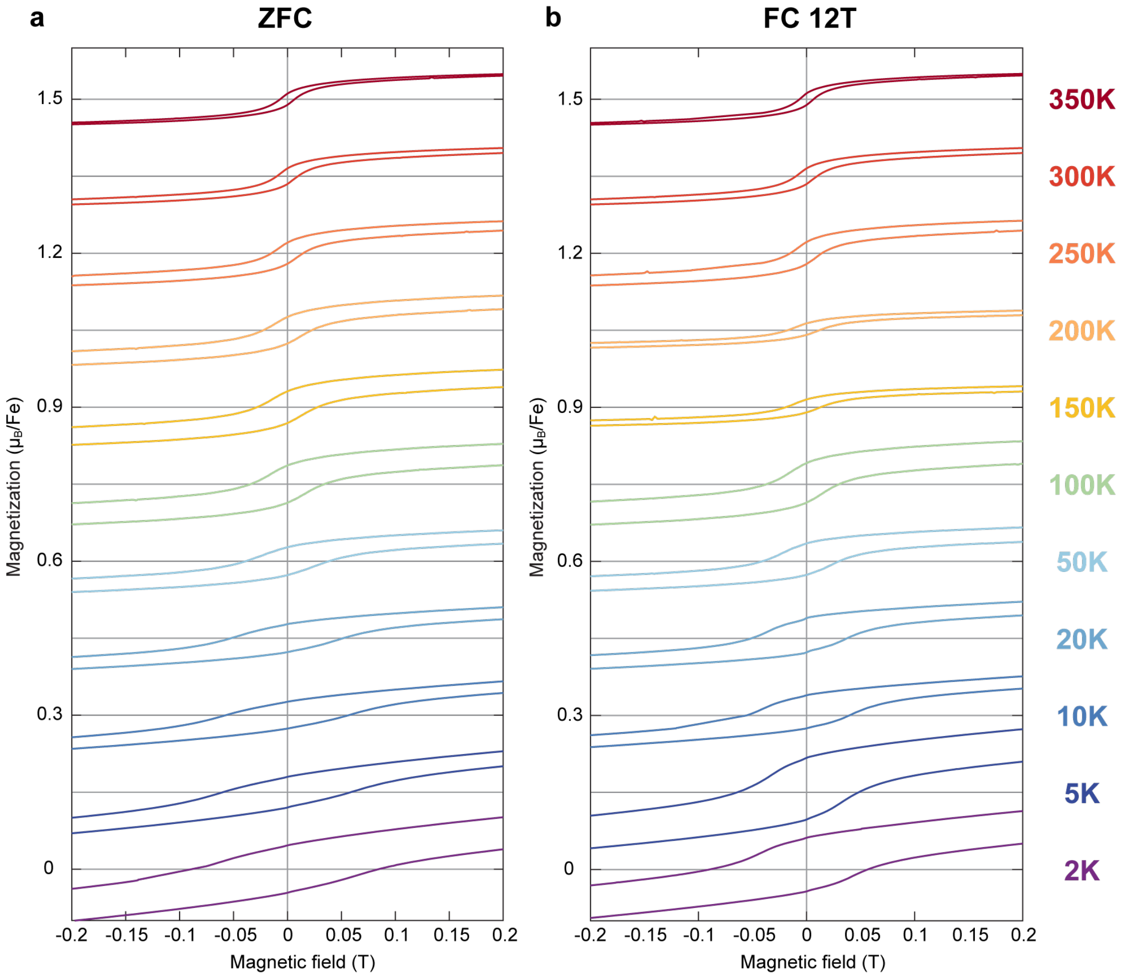

Figure S16. Magnetization versus magnetic field measurements at different temperatures. (a) ZFC and (b) FC under a 12 T external field and then sweep field from +12 T to −12 T back to +12 T. The applied field range of −0.2 T to 0.2 T is presented here. Each loop is offset on the y-axis by 0.15 μ_B/Fe. The magnetic field was applied along the c-axis of the samples.

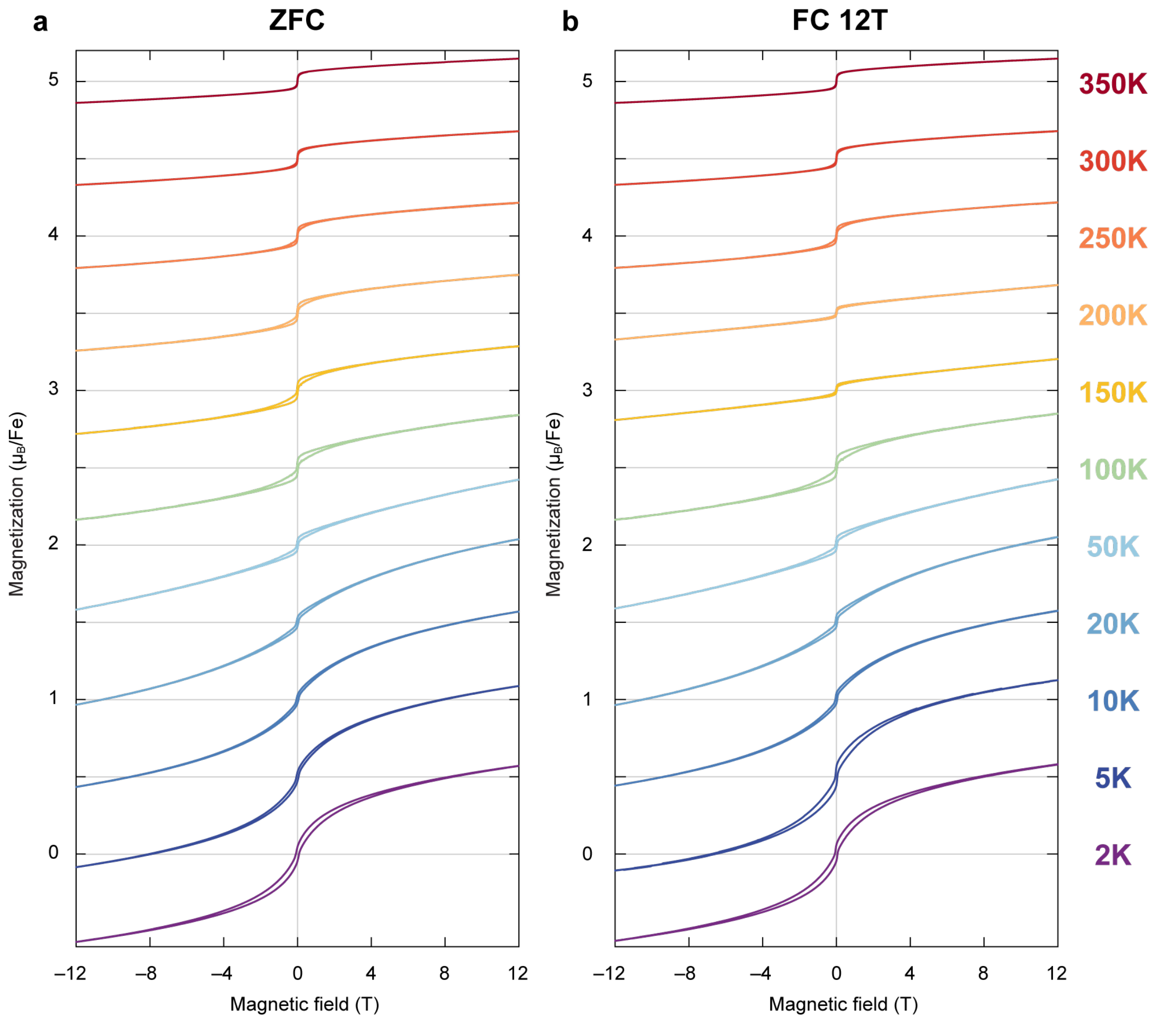

Figure S17. Magnetization versus magnetic field measurements at different temperatures. Same as Figure S16 but presented with the full range of applied field. Each loop is offset on the y-axis by 0.5 μ_B/Fe.

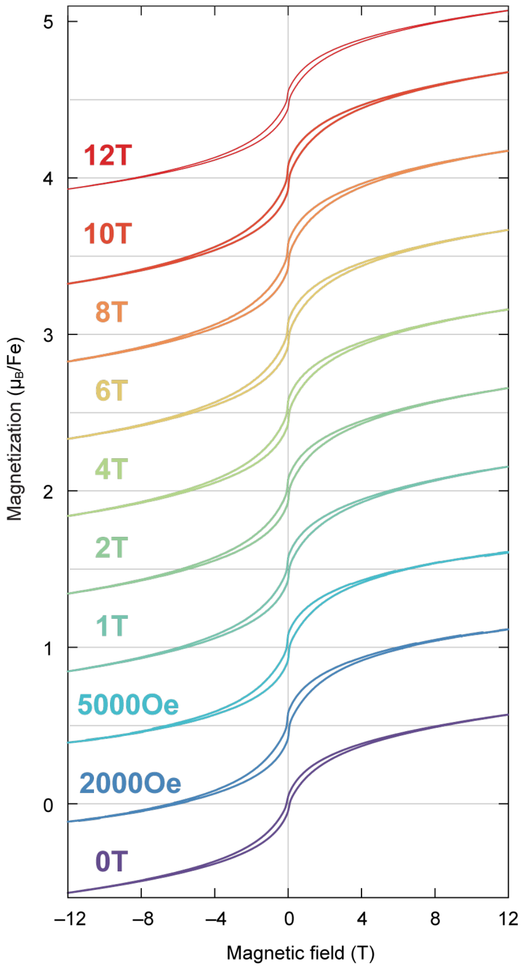

Figure S18. Magnetization versus magnetic field measurements at 2K with sample cooled down in different cooling fields. Each loop is offset on the y-axis by 0.5 μ_B/Fe The magnetic field was applied along the c-axis of the samples.

S19. Steric effect on the occupation of Fe in vdWs gap

To gain insights into the impact of steric effects on complex stability, we compare the sizes of octahedra and tetrahedra in iron selenide compounds and Fe-intercalated TMDs. We examined the reported crystal structures for $FeSe_x$ compounds and Fe_xMSe_2 (M = transition metal) and extracted the Se–Se distances of octahedra (**Table S5**) and tetrahedra (**Table S6**). Compositions without published crystal structures are marked with a "–". The extracted Se–Se distances of octahedra are plotted in **Figure S19b** and those of tetrahedra are presented in **Figure S19c**.

This simple analysis shows that the sizes of the $FeSe_6$ octahedra in Fe_xMSe_2 compounds are within the range of those found in $FeSe_x$ compounds. However, we find that the sizes of the $FeSe_4$ tetrahedra in Fe_xMSe_2 compounds are consistently smaller than those found in FeSe, but Fe_xZrSe_2, possesses the $FeSe_4$ tetrahedron that is closest to that found in FeSe. This comparison hints to the reason why Zr-based TMDs are uniquely capable of accommodating a significant fraction of Fe intercalated in tetrahedral sites. Further theoretical investigations are required to gain a deeper understanding of the influence of steric effects on the preferred intercalation sites of Fe.

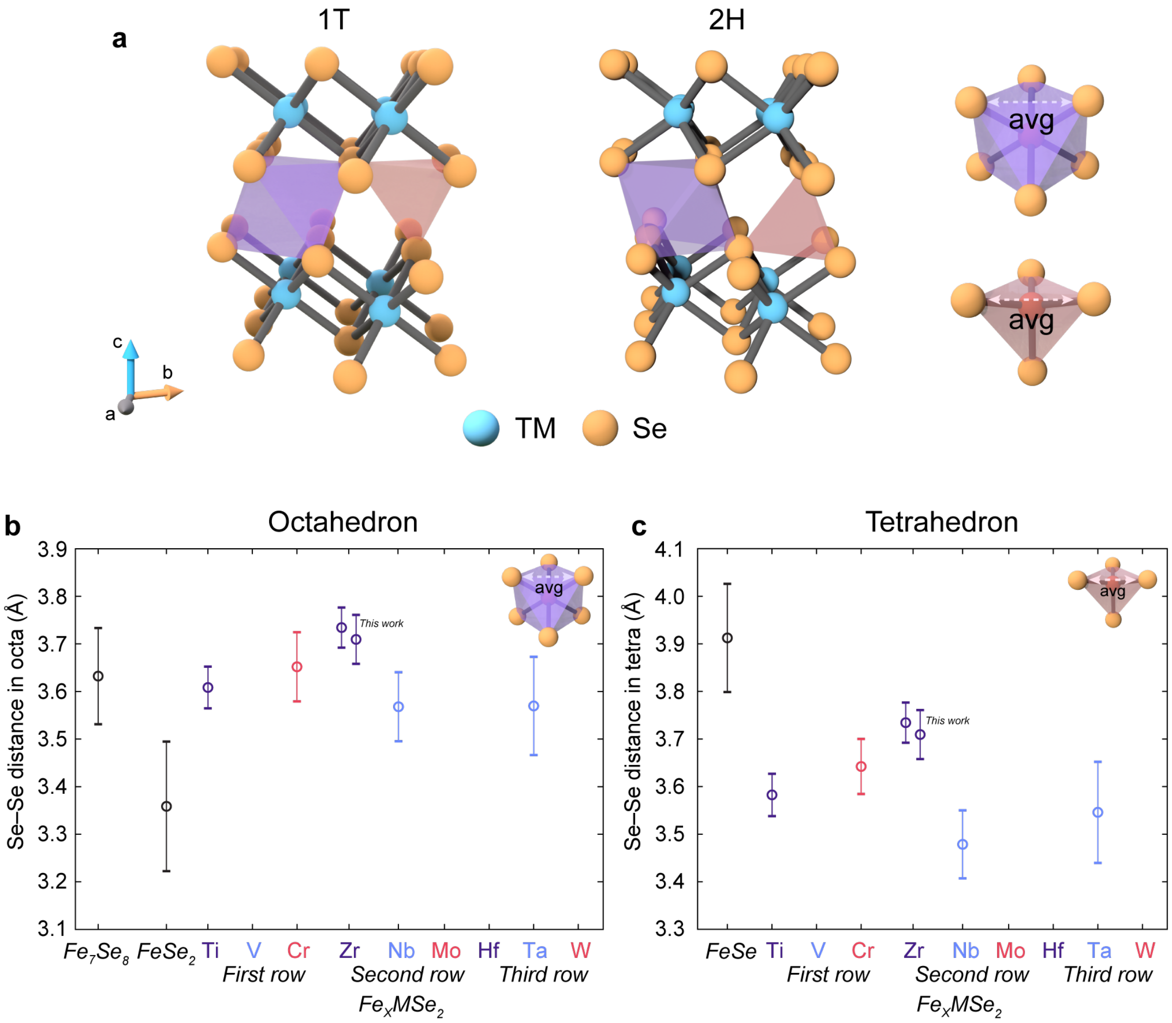

Figure S19. The comparison of Se−Se distances for $FeSe_4$ tetrahedra and $FeSe_6$ octahedra in $FeSe_x$ compounds versus in Fe intercalated TMDs.

Table S5. Se−Se distance for $FeSe_6$ octahedra

	Average Se−Se distance (Å)	Standard deviation Se−Se distance (Å)	ref
$FeSe_2$	3.36	0.14	23-28
Fe_7Se_8	3.63	0.10	29
Fe_xTiSe_2	3.61	0.04	30-33
Fe_xVSe_2	–	–	
Fe_xCrSe_2	3.65	0.07	34-36
Fe_xZrSe_2	3.73	0.04	37-38
Fe_xNbSe_2	3.57	0.07	39
Fe_xMoSe_2	–	–	
Fe_xHfSe_2	–	–	
Fe_xTaSe_2	3.57	0.10	40
Fe_xWSe_2	–	–	
Fe_xZrSe_2 (This work)	3.71	0.05	

Table S6. Se−Se distances in $FeSe_4$ tetrahedra

	Average Se−Se distance (Å)	Standard deviation Se−Se distance (Å)	ref
FeSe	3.91	0.11	41-48
Fe_xTiSe_2	3.58	0.04	30-33
Fe_xVSe_2	×	×	
Fe_xCrSe_2	3.64	0.06	34-36
Fe_xZrSe_2	3.73	0.04	37-38
Fe_xNbSe_2	3.48	0.07	39
Fe_xMoSe_2	×	×	
Fe_xHfSe_2	×	×	
Fe_xTaSe_2	3.55	0.11	40
Fe_xWSe_2	×	×	
Fe_xZrSe_2 (This work)	3.71	0.05	

S20. The contextualization of representative exchange bias systems

Table S7. Representative exchange bias systems

Structure	Materials	Magnetic phase	largest H_{EB}	Temp (largest H_{EB})	ref
Intrinsic exchange bias	$YbFe_2O_4$	AFM/FM two magnetic sublattices	19 kOe	5 K	49
	Aurivillius oxides $Bi_{10}Fe_{6+y}Ti_{3-y}O_{30+\delta}$	coupling between AFM and spin glass	38 Oe	300 K	50
	Fe_xNbS_2 (x = 0.30, 0.35)	coupling between AFM and spin glass	30 kOe	1.8 K	51
	$Mn_3(C_6S_6)$	Geometric frustrated spin glass	1625 Oe	2 K	52
	$Fe_{0.17}ZrSe_2$	Structural disorder induced spin glass	166 Oe	2 K	This work
heterostructure	Co/CoO	Ferromagnet/ Ferromagnet	9500 Oe	4-10 K	53
	Co/CoN		3200 Oe	4-10 K	
	Ni/NiO	Ferromagnet/ antiferromagnet	400 Oe	4-10 K	
	Fe/Fe_3O_4	Ferromagnet/ Ferrimagnet/	120 Oe	4-10 K	
	Fe/Fe_2N	Ferromagnet/ Ferromagnet	300 Oe	4-10 K	
	Co/CuMn	Ferromagnet/spin glass	85 Oe	2 K	54

S21. Reference:

1. Veith, G. M.; Chen, R. J.; Popov, G.; Croft, M.; Shokh, Y.; Nowik, I.; Greenblatt, M. Electronic, magnetic, and magnetoresistance properties of the n=2 Ruddlesden-Popper phases $Sr_3Fe_{2-x}CO_xO_{7-\delta}$ (0.25 <= x <= 1.75). *J. Solid State Chem.* **2002**, *166*, 292–304.
2. Joseph, B.; Iadecola, A.; Simonelli, L.; Mizuguchi, Y.; Takano, Y.; Mizokawa, T.; Saini, N. L. A study of the electronic structure of $FeSe_{1-x}Te_x$ chalcogenides by Fe and Se K-edge x-ray absorption near edge structure measurements. *J Phys-Condens Mat* **2010**, *22*, 485702.
3. Xie, L. S.; Husremovic, S.; Gonzalez, O.; Craig, I. M.; Bediako, D. K. Structure and Magnetism of Iron- and Chromium-Intercalated Niobium and Tantalum Disulfides. *J. Am. Chem. Soc.* **2022**, *144*, 9525–9542.
4. Mydosh, J. A. *Spin glasses: an experimental introduction*; 1st ed.; Taylor & Francis, London ; Washington, DC, 1993.
5. Ueno, K. Introduction to the Growth of Bulk Single Crystals of Two-Dimensional Transition-Metal Dichalcogenides. *J. Phys. Soc. Jpn.* **2015**, *84*, 121015.
6. Husremović, S.; Groschner, C. K.; Inzani, K.; Craig, I. M.; Bustillo, K. C.; Ercius, P.; Kazmierczak, N. P.; Syndikus, J.; Van Winkle, M.; Aloni, S.; Taniguchi, T.; Watanabe, K.; Griffin, S. M.; Bediako, D. K. Hard Ferromagnetism Down to the Thinnest Limit of Iron-Intercalated Tantalum Disulfide. *J. Am. Chem. Soc.* **2022**, *144*, 12167–12176.
7. Sheldrick, G. M. A short history of SHELX. *Acta Cryst. A* **2008**, *64*, 112–122.
8. Sheldrick, G. M., SHELXS-2014: Program for the Solution of Crystal Structures. University Of Göttingen: 2014.
9. Sheldrick, G. M. Crystal structure refinement with SHELXL. *Acta Cryst. C* **2015**, *71*, 3–8.
10. Sheldrick, G. M., SHELXL-2014: Crystallographic Software Package. Bruker AXS, Inc.: Madison, WI: 2014.
11. Dolomanov, O. V. a. B., Luc J. and Gildea, Richard J. and Howard, Judith A. K. and Puschmann, Horst OLEX2: a complete structure solution, refinement and analysis program. *J. Appl. Crystallogr.* **2009**, *42*, 339–341.
12. Momma, K.; Izumi, F. VESTA 3 for three-dimensional visualization of crystal, volumetric and morphology data. *J. Appl. Crystallogr.* **2011**, *44*, 1272–1276.
13. Wong, J.; Lytle, F. W.; Messmer, R. P.; Maylotte, D. H. K-Edge Absorption-Spectra of Selected Vanadium Compounds. *Phys. Rev. B* **1984**, *30*, 5596–5610.
14. Zhu, J.; Zeng, Z. H.; Li, W. X. K-Edge XANES Investigation of Fe-Based Oxides by Density Functional Theory Calculations. *J. Phys. Chem. C* **2021**, *125*, 26229–26239.
15. Kim, M. G.; Cho, H. S.; Yo, C. H. Fe K-edge X-ray absorption (XANES/EXAFS) spectroscopic study of the nonstoichiometric $SrFe_{1-x}Sn_xO_{3-y}$ system. *J. Phys. Chem. Solids* **1998**, *59*, 1369–1381.
16. Brauer, H. E.; Starnberg, H. I.; Holleboom, L. J.; Hughes, H. P. The Electronic-Structure of $ZrSe_2$ and Cs_xZrSe_2 Studied by Angle-Resolved Photoelectron-Spectroscopy. *J Phys-Condens Mat* **1995**, *7*, 7741–7760.
17. Kubelka, P. M., Franz A contribution to the optics of pigments. *Z. Tech. Phys.* **1931**, *12*, 593–599.
18. Tauc, J. Optical properties and electronic structure of amorphous Ge and Si. *Mater. Res. Bull.* **1968**, *3*, 37–46.
19. Lopez, R.; Gomez, R. Band-gap energy estimation from diffuse reflectance measurements on sol-gel and commercial TiO_2: a comparative study. *J. Sol-Gel Sci. Technol.* **2012**, *61*, 1–7.
20. Tong, P.; Sun, Y. P.; Zhu, X. B.; Song, W. H. Strong electron-electron correlation in the antiperovskite compound $GaCNi_3$. *Phys. Rev. B* **2006**, *73*, 245106.
21. Goetsch, R. J.; Anand, V. K.; Pandey, A.; Johnston, D. C. Structural, thermal, magnetic, and electronic transport properties of the $LaNi_2(Ge_{1-x}P_x)_2$ system. *Phys. Rev. B* **2012**, *85*, 054517.
22. Buhannic, M. A.; Danot, M.; Colombet, P.; Dordor, P.; Fillion, G. Thermopower and Low-DC-Field Magnetization Study of the Layered Fe_xZrSe_2 Compounds - Anderson-Type Localization and Anisotropic Spin-Glass Behavior. *Phys. Rev. B* **1986**, *34*, 4790–4795.

23. Tengner, S. Über Diselenide und Ditelluride von Eisen, Kobalt und Nickel. *Z. Anorg. Allg. Chem.* **1938**, *239*, 126–132.
24. Schuster, W.; Mikler, H.; Komarek, K. L. Transition metal-chalcogen systems, VII.: The iron-selenium phase diagram. *Monatshefte für Chemie / Chemical Monthly* **1979**, *110*, 1153–1170.
25. Pickardt, J.; Reuter, B.; Riedel, E.; Söchtig, J. On the formation of $FeSe_2$ single crystals by chemical transport reactions. *J. Solid State Chem.* **1975**, *15*, 366–368.
26. Buryanova, E. Z. K., A.I. A new mineral—Ferroselite. *Doklady Akademii Nauk SSSR* **1955**, *105*, 812–813.
27. Arne Kjekshus, T. R. Coumpounds with the Marcasite Type Crystal Structure. XI. High Temperature Studies of Chalcogenides. *Acta Cryst. A* **1975**, *29*, 443–452.
28. Andresen, A. K. T. R. A. F. Compounds with the Marcasite Type Crystal Structure. IX. Structural Data for $FeAs_2$, $FeSe_2$, $NiAs_2$, $NiSb_2$, and $CuSe_2$. *Acta Chem. Scand.* **1974**, *28*, 996–1000.
29. Parise, J. B.; Nakano, A.; Tokonami, M.; Morimoto, N. Structure of iron selenide 3*C*-Fe_7Se_8. *Acta Cryst. B* **1979**, *35*, 1210–1212.
30. Shkvarina, E. G.; Titov, A. A.; Shkvarin, A. S.; Postnikov, M. S.; Radzivonchik, D. I.; Plaisier, J. R.; Gigli, L.; Gaboardi, M.; Titov, A. N. Thermal disorder in the $Fe_{0.5}TiSe_2$. *J. Alloys Compd.* **2020**, *819*, 153016.
31. Lyding, J. W.; Ratajack, M. T.; Kannewurf, C. R.; Goodman, W. H.; Ibers, J. A.; Marsh, R. E. Structure, Electrical Transport, and Optical-Properties of a New Ordered Iron Intercalated Dichalcogenide, $Fe_{0.34}TiSe_2$. *J. Phys. Chem. Solids* **1982**, *43*, 599–607.
32. Huntley, D. R.; Sienko, M. J.; Hiebl, K. Magnetic-Properties of Iron-Intercalated Titanium Diselenide. *J. Solid State Chem.* **1984**, *52*, 233–243.
33. Calvarin, G.; Gavarri, J. R.; Buhannic, M. A.; Colombet, P.; Danot, M. Crystal and Magnetic-Structures of $Fe_{0.25}TiSe_2$ and $Fe_{0.48}TiSe_2$. *Revue De Physique Appliquee* **1987**, *22*, 1131–1138.
34. Riedel, E.; Al-Juani, A.; Rackwitz, R.; Söchtig, H. Spinelle mit substituierten Nichtmetallteilgittern. VIII. Röntgenographische und elektrische Eigenschaften, Mößbauer- und IR-Spektren des Systems $FeCr_2(S_{1-x}Se_x)_4$. *Z. Anorg. Allg. Chem.* **1981**, *480*, 49–59.
35. Kim, J. H. K. S. J. K. B. W. L. C. S. Neutron and Mössbauer studies of $FeCr_2Se_4$. *J. Appl. Phys.* *99*, 08F714.
36. Morris, B. L.; Russo, P.; Wold, A. Magnetic properties of ACr_2Se_4 (A = Fe, Co, Ni) and $NiCr_2S_4$. *J. Phys. Chem. Solids* **1970**, *31*, 635–638.
37. Buhannic, M.-A.; Ahouandjinou, A.; Danot, M.; Rouxel, J. Double coordinence du fer dans la phase Fe_xZrSe_2 (0 < x < 0,25): propriétés magnétiques et caractéristiques Mössbauer. *J. Solid State Chem.* **1983**, *49*, 77–84.
38. Ahouandjinou, A. T., Luc; Rouxel, Jean Chimie minérale. Les phases Fe_xZrSe_2 (0< x< 0.25), Co_xZrSe_2 (0< x< 0.33) et Ni_xZrSe_2 (0< x< 0.50). *Comptes Rendus des Seances de l'Academie des Sciences, Serie C: Sciences Chimiques* **1976**, 727–730.
39. Erodici, M. P.; Mai, T. T.; Xie, L. S.; Li, S.; Fender, S. S.; Husremović, S.; Gonzalez, O.; Hight Walker, A. R.; Bediako, D. K. Bridging Structure, Magnetism, and Disorder in Iron-Intercalated Niobium Diselenide, Fe_xNbSe_2, below x = 0.25. *J. Phys. Chem. C* **2023**.
40. Mrotzek, B. H. A. Synthese, Struktur und Eigenschaften ternaerer Tantalselenide. *Zeitschrift für Kristallographie. Supplement issue* **1995**, 169.
41. Pomjakushina, E.; Conder, K.; Pomjakushin, V.; Bendele, M.; Khasanov, R. Synthesis, crystal structure, and chemical stability of the superconductor $FeSe_{1-x}$. *Phys. Rev. B* **2009**, *80*, 024517.
42. Li, Z. F.; Ju, J.; Tang, J.; Sato, K.; Watahiki, M.; Tanigaki, K. Structural and superconductivity study on α-$FeSe_x$. *J. Phys. Chem. Solids* **2010**, *71*, 495–498.
43. Horigane, K.; Hiraka, H.; Ohoyama, K. Relationship between Structure and Superconductivity in $FeSe_{1-x}Te_x$. *J. Phys. Soc. Jpn.* **2009**, *78*, 074718.
44. Millican, J. N.; Phelan, D.; Thomas, E. L.; Leao, J. B.; Carpenter, E. Pressure-induced effects on the structure of the FeSe superconductor. *Solid State Commun.* **2009**, *149*, 707–710.

45. Margadonna, S.; Takabayashi, Y.; Ohishi, Y.; Mizuguchi, Y.; Takano, Y.; Kagayama, T.; Nakagawa, T.; Takata, M.; Prassides, K. Pressure evolution of the low-temperature crystal structure and bonding of the superconductor FeSe (T_C=37 K). *Phys. Rev. B* **2009**, *80*, 064506.
46. McQueen, T. M.; Huang, Q.; Ksenofontov, V.; Felser, C.; Xu, Q.; Zandbergen, H.; Hor, Y. S.; Allred, J.; Williams, A. J.; Qu, D.; Checkelsky, J.; Ong, N. P.; Cava, R. J. Extreme sensitivity of superconductivity to stoichiometry in $Fe_{1+\delta}Se$. *Phys. Rev. B* **2009**, *79*, 014522.
47. Margadonna, S.; Takabayashi, Y.; McDonald, M. T.; Kasperkiewicz, K.; Mizuguchi, Y.; Takano, Y.; Fitch, A. N.; Suard, E.; Prassides, K. Crystal structure of the new $FeSe_{1-x}$ superconductor. *Chem. Commun.* **2008**, *43*, 5607–5609.
48. Kumar, R. S.; Zhang, Y.; Sinogeikin, S.; Xiao, Y. M.; Kumar, S.; Chow, P.; Cornelius, A. L.; Chen, C. F. Crystal and Electronic Structure of FeSe at High Pressure and Low Temperature. *J. Phys. Chem. B* **2010**, *114*, 12597–12606.
49. Sun, Y.; Cong, J. Z.; Chai, Y. S.; Yan, L. Q.; Zhao, Y. L.; Wang, S. G.; Ning, W.; Zhang, Y. H. Giant exchange bias in a single-phase magnet with two magnetic sublattices. *Appl. Phys. Lett.* **2013**, *102*, 172406.
50. Wang, G. P.; Chen, Z. Z.; He, H. C.; Meng, D. C.; Yang, H.; Mao, X. Y.; Pan, Q.; Chu, B. J.; Zuo, M.; Sun, Z. H.; Peng, R. R.; Fu, Z. P.; Zhai, X. F.; Lu, Y. L. Room Temperature Exchange Bias in Structure-Modulated Single-Phase Multiferroic Materials. *Chem. Mater.* **2018**, *30*, 6156–6163.
51. Maniv, E.; Murphy, R. A.; Haley, S. C.; Doyle, S.; John, C.; Maniv, A.; Ramakrishna, S. K.; Tang, Y. L.; Ercius, P.; Ramesh, R.; Reyes, A. P.; Long, J. R.; Analytis, J. G. Exchange bias due to coupling between coexisting antiferromagnetic and spin-glass orders. *Nat. Phys.* **2021**, *17*, 525–530.
52. Murphy, R. A.; Darago, L. E.; Ziebel, M. E.; Peterson, E. A.; Zaia, E. W.; Mara, M. W.; Lussier, D.; Velasquez, E. O.; Shuh, D. K.; Urban, J. J.; Neaton, J. B.; Long, J. R. Exchange Bias in a Layered Metal-Organic Topological Spin Glass. *ACS Cent. Sci.* **2021**, *7*, 1317–1326.
53. Nogués, J.; Schuller, I. K. Exchange bias. *J. Magn. Magn. Mater.* **1999**, *192*, 203–232.
54. Ali, M.; Adie, P.; Marrows, C. H.; Greig, D.; Hickey, B. J.; Stamps, R. L. Exchange bias using a spin glass. *Nat. Mater.* **2007**, *6*, 70–75.
